# Integrating Plasma mNGS into ICU Antimicrobial Stewardship: A Four-Phase Framework

**DOI:** 10.3390/antibiotics15100978

**Published:** 2026-10-03

**Authors:** Torsten H. Schroeder, Mamoun Eliwi Alsaffan, Angelina Bondarenko, Albion Dervishi

**Affiliations:** 1 Department of Anesthesiology and Intensive Care Medicine, Medius KLINIK Nuertingen, Academic Teaching Hospital of the University Hospital Tuebingen, 72622 Nuertingen, Germany; 2 Department of Internal and Emergency Medicine, Medius KLINIK Nuertingen, Academic Teaching Hospital of the University Hospital Tuebingen, 72622 Nuertingen, Germany

**Keywords:** metagenomic next-generation sequencing (mNGS), antimicrobial stewardship, intensive care unit, blood culture, biomarker kinetics, procalcitonin, sepsis

## Abstract

**Background:** Plasma metagenomic next-generation sequencing (mNGS) may complement conventional microbiology in critically ill patients, yet its contribution to antimicrobial stewardship in the intensive care unit (ICU) remains unclear. **Methods:** In this retrospective, single-center observational study, we analyzed 243 paired mNGS–blood culture episodes from 180 ICU patients. We applied a four-phase stewardship framework that integrated clinical severity, biomarker kinetics, blood culture results, mNGS findings, and clinical assessment. Treatment adaptations were classified as escalation, de-escalation, discontinuation, or no change. Multivariable regression identified factors associated with stewardship modification and mortality. **Results:** Active treatment adaptation occurred in 121 of 243 episodes (49.8%), comprising 61 escalations, 39 de-escalations, and 21 discontinuations. mNGS positivity was independently associated with treatment modification (OR 2.66, 95% CI 1.23–5.75; *p* = 0.014), whereas blood culture positivity showed an inverse association (OR 0.45, 95% CI 0.20–1.04; *p* = 0.062). Mortality was primarily linked to physiologic severity and host-response markers, particularly SAPS II and procalcitonin. In-hospital survival did not differ between patients who received stewardship-guided adaptation and those in whom empirical antibiotic therapy was maintained (HR 1.02, 95% CI 0.57–1.81; log-rank *p* = 0.951). **Conclusions:** Microbiological findings, particularly mNGS, were associated with antimicrobial treatment adaptation but not independently with mortality. Because mNGS was embedded within a multimodal diagnostic and clinical pathway, its independent contribution cannot be fully separated from concurrent microbiological, biomarker, and clinical information. These findings should be regarded as exploratory and require prospective multicenter validation.

## 1. Introduction

Empirical antibiotic therapy remains the standard first-line approach in the ICU, yet blood culture identifies the pathogen in only a fraction of patients and takes one to two days to report [1]. This diagnostic gap forces clinicians to choose between prolonged broad-spectrum coverage—with attendant risks of resistance selection and toxicity—and premature narrowing based on incomplete information [2].

Metagenomic next-generation sequencing (mNGS) can detect a wide range of pathogens, including anaerobes, that conventional culture often misses [3]. But its real-world impact on treatment decisions is inconsistent. Some studies show that earlier mNGS-guided changes reduce mortality [4]. Others show mNGS changes treatment in only a small number of patients, mainly because results come back too late [5]. Most studies also look at mNGS as a single decision at one time point, not as part of an ongoing process [4,5].

Unlike blood culture, plasma mNGS detects microbial cell-free DNA (cfDNA) without requiring organism growth. It may therefore retain diagnostic yield during antimicrobial therapy and can identify non-culturable pathogens [6,7]. However, microbial DNA detection does not establish organism viability or causality, and low-level signals may represent background DNA, transient DNAemia, or contamination [8].

No formal guidelines currently exist for integrating plasma mNGS into ICU antimicrobial stewardship. Consequently, clinicians must interpret mNGS findings alongside blood culture results, biomarker kinetics, and the overall clinical context [7]. How plasma mNGS contributes to antimicrobial stewardship decisions in routine ICU practice remains poorly defined.

To address this knowledge gap, we developed a four-phase stewardship framework that conceptualizes antimicrobial stewardship as a continuous process, integrating admission severity, biomarker kinetics, blood culture findings, and plasma mNGS as diagnostic information became available. Using this framework, we retrospectively evaluated the frequency and pattern of mNGS-guided antimicrobial stewardship adaptations and identified the clinical determinants associated with stewardship decision-making.

## 2. Methods

### 2.1. Study Design and Population

This retrospective observational single-center study included adult intensive care unit (ICU) patients (≥18 years) who underwent plasma metagenomic next-generation sequencing (mNGS) testing between March 2023 and September 2024 at a tertiary mixed ICU. The dataset used in this work derives from our previously described mNGS cohort [3].

From 393 paired mNGS–blood culture sampling episodes obtained from 302 unique ICU patients, 150 episodes were excluded from the stewardship analysis. Exclusions comprised 36 episodes from patients who died before microbiological results became available, 13 episodes from patients transferred before results were available, 4 episodes with early discharge, and 97 episodes without an evaluable stewardship decision (82 with no actionable signal [mNGS−/BC−/Abx−] and 15 with missing documentation of the stewardship decision). The final analytic cohort therefore consisted of 243 sampling episodes from 180 unique patients (Figure 1). In a sensitivity analysis, in-hospital and ICU mortality among patients with non-evaluable episodes did not differ significantly from the analytic cohort (32.4% vs. 26.1%, *p* = 0.36; 17.6% vs. 11.1%, *p* = 0.22; Supplementary Table S2).

Blood cultures (BC) and plasma mNGS testing were ordered in parallel by treating clinicians at the time of ICU admission. Empirical broad-spectrum antibiotic therapy was not protocolized, as treatment had frequently been initiated on a referring ward or intraoperatively prior to ICU admission.

At our institution, BC results were available continuously; reporting time depended on growth kinetics but was typically one to two days. mNGS results became available between Day 2 and Day 4 and were subsequently used to guide decisions regarding antibiotic escalation, de-escalation, continuation, or discontinuation. Episodes were classified into diagnostic strata according to BC and mNGS results and antibiotic exposure status at the time of sampling. Empirical antimicrobial therapy was initiated when clinically indicated on the basis of suspected infection.

Collected variables included patient demographics, admission severity parameters, the Simplified Acute Physiology Score II (SAPS II), inflammatory biomarkers (C-reactive protein [CRP], procalcitonin [PCT], leukocyte count, and body temperature), microbiological findings from blood culture and mNGS, antibiotic therapy and stewardship modifications, and ICU mortality outcomes.

The study was conducted in accordance with the Declaration of Helsinki and was approved by the Ethics Committee of the Medical Faculty of the University of Tübingen (487/2024BO2).

### 2.2. Metagenomic Next-Generation Sequencing

The plasma mNGS workflow followed our previously reported protocol [3]. Whole blood (5–10 mL) was drawn into Streck Cell-Free DNA BCT tubes (Streck Inc., La Vista, NE, USA) and analyzed at a dedicated reference laboratory (Labor Dr. Wisplinghoff, Cologne, Germany). Following a two-step centrifugation, circulating cell-free DNA was extracted with the QIAsymphony DSP Circulating DNA Kit (QIAGEN GmbH, Hilden, Germany). Sequencing libraries were generated from 2 ng of input cfDNA with the NEXTFLEX^®^ Cell Free DNA-Seq Library Prep Kit 2.0 (Revvity Inc., Waltham, MA, USA) and run on a NextSeq 550 instrument (Illumina Inc., San Diego, CA, USA) with a NextSeq 500/550 Mid Output Kit. Sequence-based pathogen identification relied on the CE-IVD-cleared DISQVER^®^ platform (Noscendo GmbH, Duisburg, Germany) running DISQVER^®^ R6 software.

The DISQVER^®^ pipeline applies proprietary bioinformatic filtering and reference-database matching to identify and quantitatively report microbial cfDNA. Exact internal significance thresholds are not publicly disclosed by the manufacturer. Effective scaled read counts were additionally analyzed by microbial category, as detailed in Supplementary Material S1.

### 2.3. Four-Phase mNGS-Guided Antibiotic Stewardship Model

A four-phase stewardship framework was constructed retrospectively to classify antibiotic decisions made during routine clinical care. The framework integrates admission severity, biomarker kinetics, microbiological findings, and clinical response into a structured model for antibiotic decision support. Antibiotic decisions were made by the treating ICU team during structured morning rounds and reflected multidisciplinary, team-based clinical judgment. Stewardship decisions were not retrospectively re-rated or adjudicated. They were extracted from contemporaneous documentation in the electronic health record (ORBIS) by two co-authors, and the four-phase framework was applied only to structure these documented decisions over time (Supplementary Material S2).

Phase A (Day 0) established the baseline using CRP, PCT, leukocyte count, temperature, SAPS II, antibiotic exposure, and suspected infection source. Phase B (Days 1–2) evaluated treatment response through biomarker kinetics and sequential microbiological findings from blood cultures, enabling dynamic antibiotic stewardship decisions as new diagnostic information accrued.

Phase C (Days 3–4) represented the late stewardship phase, in which mNGS findings were integrated with clinical trajectory and blood culture results to refine antimicrobial therapy—through escalation, de-escalation, continuation, or discontinuation—as the clinical picture consolidated. Phase D captured clinical outcome, including ICU mortality and hospital survival (Figure 2).

### 2.4. Longitudinal Biomarker Trajectories and Change-from-Baseline Analysis

To characterize temporal patterns in relation to stewardship decisions, CRP, leukocyte count, PCT, SAPS II, and temperature were analyzed as continuous variables from Day 1 (baseline) through Day 5 (Figure 3). Daily values were summarized by stewardship action (no change, escalation, de-escalation, or stop) and presented as means with standard errors.

Changes from Day 1 were calculated for the Day 1→2, Day 1→3, Day 1→4, and Day 1→5 observation windows. Percentage change was used for CRP, leukocyte count, PCT, and SAPS II, whereas temperature change was expressed as the absolute difference in °C because of its narrow physiological range. Changes were summarized as medians with interquartile ranges, and the number of contributing patients was reported for each window.

Mortality discrimination was evaluated separately for each biomarker and observation window using either the value recorded at the end of the window or its change from Day 1. Discrimination for ICU mortality was quantified by the area under the receiver operating characteristic curve (AUC) with 95% confidence intervals. These exploratory analyses compared the prognostic information provided by observed biomarker values with that provided by longitudinal changes. Although literature-based cutoffs have been proposed for PCT and CRP [9,10,11] and biologically motivated rationale exists for the directionality of temperature and leukocyte changes [12,13] the present model relied solely on continuous change scores.

### 2.5. Statistical Analysis

Continuous variables are presented as median [IQR] and categorical variables as counts (%). Group comparisons were performed using the Wilcoxon rank-sum test for continuous variables and Fisher’s exact test for categorical variables. Standardized mean differences were additionally reported for selected baseline comparisons. Multivariable logistic regression evaluated factors associated with hospital and ICU mortality, reported as odds ratios (ORs) with 95% confidence intervals. Covariates were selected a priori according to the four-phase framework (Section 2.3), without data-driven selection. Phase A included age, sex, SAPS II, and baseline antibiotic exposure; Phase B added early biomarker kinetics and blood culture results; and Phase C added mNGS results. The number of predictors was limited within each phase to avoid overfitting given the number of outcome events. For hospital mortality (47 events/180 patients), Firth penalized logistic regression was used; for ICU mortality, it was applied as a sensitivity analysis because of the limited event count (detailed results are provided in Supplementary Material S1). Absolute biomarker values and changes from baseline were modeled separately to reduce collinearity. The stewardship-decision model (121 adapted episodes out of 243) was restricted to prespecified clinically relevant predictors. Missing data were handled by complete-case analysis for each model, with sample size varying according to biomarker availability; no imputation was performed. Biomarker discrimination was explored using receiver operating characteristic (ROC) analysis. For survival analysis, time zero was defined as the date of the index mNGS/blood-culture sampling episode. Patients were followed from index sampling until in-hospital death or hospital discharge, with patients discharged alive censored at discharge. Survival distributions were estimated using Kaplan–Meier methods and compared using the log-rank test; Cox proportional-hazards models were used to estimate hazard ratios with 95% confidence intervals. All analyses were exploratory, and no formal adjustment for multiple comparisons was performed. Two-sided *p* < 0.05 was considered statistically significant. Analyses were performed in R version 4.5.3 using the tidyverse, pROC, survival, survminer, and logistf packages. OpenAI tools were used solely to assist with debugging R code.

## 3. Results

### 3.1. Cohort Characteristics

The final analytic dataset comprised 243 sampling episodes from 180 unique ICU patients. At the patient level, empirical therapy was maintained in 86 patients, whereas 94 patients underwent at least one stewardship-guided antimicrobial adaptation during the analytic period. Baseline demographic and comorbidity characteristics were comparable between groups: median age was 73.0 years in both (*p* = 0.166; SMD = 0.26).

Sex distribution, admission SAPS II, and baseline inflammatory/biochemical markers (CRP, PCT, leukocytes, temperature, creatinine) did not differ significantly between groups, nor did the prevalence of comorbidities (arterial hypertension, cardiovascular disease, pulmonary disease, renal disease, prior stroke, and diabetes mellitus) (Table 1A,B).

At the patient level, ICU length of stay was longer with stewardship-guided adaptation (12.0 [6.0, 24.0] vs 7.0 [4.0, 15.0] days; *p* = 0.003; SMD = 0.46), while hospital length of stay was similar (23.0 [12.0, 33.0] vs 23.5 [12.0, 40.0] days; *p* = 0.480; SMD = 0.11).

ICU mortality was 9.3% vs 12.8% (*p* = 0.487; SMD = 0.11), and hospital mortality was 24.4% vs 27.7% (*p* = 0.734; SMD = 0.07) (Table 2).

### 3.2. Antimicrobial Treatment Adaptations According to mNGS and Blood Culture Results

Overall, antimicrobial stewardship adaptations were executed in 121 of 243 sampling episodes (49.8%). Episodes were stratified according to mNGS result, blood culture (BC) result, and antibiotic exposure at the time of sampling (Abx+/Abx−) (Table 3). Stewardship decisions were classified as no change, escalation, de-escalation, or discontinuation of antibiotic therapy. The largest subgroup comprised mNGS-positive, BC-negative episodes obtained during ongoing antibiotic therapy (*n* = 92). In this group, active treatment adaptations were implemented in 53 episodes (57.6%), including 27 escalations, 17 de-escalations, and nine discontinuations. The second-largest subgroup comprised mNGS-positive, BC-negative episodes without antibiotic exposure at sampling (*n* = 50). In this group, therapy remained unchanged in 20 episodes, while 23 episodes underwent escalation and seven underwent de-escalation; no antibiotic discontinuations occurred. Overall, 30 of 50 episodes (60.0%) resulted in an active stewardship adaptation. Across the full cohort, escalation was the most frequent active adaptation (*n* = 61, 25.1%), followed by de-escalation (*n* = 39, 16%) and discontinuation (*n* = 21, 8.6%). Detailed examples of antimicrobial treatment modifications are provided in Supplementary Material S2.

### 3.3. Clinical Determinants of ICU Antibiotic Stewardship Decisions

Antimicrobial stewardship decisions during Phases B and C varied across empirical antimicrobial therapy, microbiological findings, infection type, and primary clinical diagnosis (Figure 4).

Escalation was most frequent in episodes in which cephalosporin/anaerobic therapy was being administered (ceftriaxone/metronidazole, *n* = 56, 30% escalated), the regimen typically used empirically at our institution for intra-abdominal infection/perforation. Among episodes in which broad-spectrum β-lactam therapy was being administered (piperacillin/tazobactam, *n* = 58, 16% escalated), escalation—guided by BC, mNGS, and clinical presentation—proceeded to Carbapenem/reserve therapy (meropenem, *n* = 2), additional antiviral therapy (*n* = 3), antifungal therapy (*n* = 2), or Gram-positive reserve therapy (*n* = 2). Among episodes in which carbapenem/reserve therapy was being administered (meropenem, *n* = 24, 25% escalated), escalation likewise proceeded to antiviral (*n* = 2) or antifungal (*n* = 2) coverage based on BC and mNGS findings.

De-escalation occurred in 29% of episodes with Gram-positive reserve therapy, 25% of episodes with carbapenem/reserve therapy, and 22% of episodes with antifungal therapy; the pooled other/rare antimicrobial category showed a higher proportion (40%) but contained only five episodes.

Among episodes without prior antibiotic therapy (*n* = 17), antimicrobial treatment was initiated in 71%, whereas 29% remained without antimicrobial therapy after stewardship review.

Among the 21 episodes in which antimicrobial therapy was discontinued (Stop), discontinuation occurred across several preceding treatment categories, including cephalosporin/anaerobic coverage, broad-spectrum β-lactam therapy, carbapenem/reserve therapy, and antifungal therapy.

Pathogen group (Figure 4B): Stewardship decisions differed across pathogen groups. Escalation was most frequent in patients with anaerobic/gut-associated pathogens (50%), *Enterococcus* spp. (49%), Enterobacterales (41%), and viral pathogens (32%). Among smaller groups, escalation was observed in 71% of *Pseudomonas* spp. episodes and 67% of rare pathogen patterns. De-escalation was highest with *Streptococcus* spp. (50%), followed by other bacteria/mixed pathogens (25%) and *Staphylococcus* spp. (24%).

Infection type (Figure 4C): Poly-bacterial infections (*n* = 50) showed equal proportions of escalation and no change (44% each), with de-escalation in 12%. Negative microbiological findings (*n* = 53) were predominantly associated with no change (64%), while treatment was discontinued in 25%. Poly-viral infections (*n* = 5) showed a high escalation proportion (60%), although this estimate was based on a small subgroup.

Main diagnosis group (Figure 4D): Stewardship decisions also varied according to the primary clinical diagnosis. Escalation was frequent in urinary infection/urosepsis (36%), intra-abdominal infection/perforation (35%), and endocarditis/bloodstream infection (30%). In sepsis/unclear infectious focus, no change was the most frequent decision (50%), followed by de-escalation (33%).

### 3.4. Antimicrobial Stewardship Strategy and Clinical Outcomes

In the 180-patient analytic cohort, empirical therapy was maintained in 86 patients, whereas 94 patients underwent at least one stewardship-guided antimicrobial adaptation during ICU treatment. Overall in-hospital mortality was 26.1% (47/180), comprising 24.4% (21/86) in the empirical-therapy-maintained group and 27.7% (26/94) in the adapted group. Overall in-hospital survival did not differ significantly between the two stewardship groups (log-rank *p* = 0.9507). The unadjusted Cox model likewise showed no statistically significant difference in the hazard of in-hospital death between patients receiving stewardship-guided therapy and those in whom empirical therapy was maintained (HR 1.02, 95% CI 0.57–1.81). Kaplan–Meier survival curves were closely aligned between groups, with substantial overlap of the 95% confidence intervals (Figure 5).

### 3.5. Sequential Predictors of Mortality Versus Predictors of Stewardship Decision-Making

To examine whether factors associated with clinical outcome also influenced antimicrobial stewardship decisions, hospital mortality and active treatment modification were analyzed separately using multivariable regression (Figure 6 and Figure 7).

(a) Predictors of hospital mortality. Hospital mortality was evaluated at the patient level using Firth penalized logistic regression across three sequential phases reflecting increasing information availability (Figure 6). In Phase A (admission), higher SAPS II was independently associated with mortality (adjusted OR 1.58 per 10-point increase, 95% CI 1.13–2.25, *p* = 0.007); age, sex, and baseline antibiotic exposure were not.
  In Phase B (early, blood-culture–guided), blood culture positivity was not associated with mortality (OR 1.37, 95% CI 0.56–3.25, *p* = 0.485), but higher PCT (OR 1.22, 95% CI 1.05–1.45, *p* = 0.010), PCT change from baseline (OR 1.52, 95% CI 1.08–2.37, *p* = 0.015), and contemporaneous SAPS II (OR 1.77 per 10-point increase, 95% CI 1.18–2.75, *p* = 0.005) were. CRP, leukocytes, temperature, and their changes from baseline were not significant.
  In Phase C (late, mNGS-guided), neither mNGS positivity (OR 0.97, 95% CI 0.41–2.36, *p* = 0.941) nor blood culture positivity (OR 1.37, 95% CI 0.56–3.25, *p* = 0.485) was associated with mortality. Mortality remained associated with PCT (OR 1.28, 95% CI 1.03–1.62, *p* = 0.022), PCT change (OR 1.49, 95% CI 1.05–2.34, *p* = 0.024), SAPS II (OR 1.72 per 10-point increase, 95% CI 1.11–2.75, *p* = 0.014), and temperature (OR 1.28, 95% CI 1.01–1.64, *p* = 0.044); CRP and leukocytes remained non-significant. Absolute and change-from-baseline biomarkers were modeled separately and are displayed together in Figure 6.
(b) Predictors of active antimicrobial stewardship decisions. A different pattern emerged when the outcome was an executed treatment modification (escalation, de-escalation, or discontinuation vs. no change) across 243 episodes from 180 patients (Figure 7A). Using logistic regression with CR2 clustered standard errors and Satterthwaite inference, mNGS positivity was independently associated with greater odds of stewardship change (OR 2.66, 95% CI 1.23–5.75, *p* = 0.014), while blood culture positivity showed a trend toward lower odds of an active stewardship change (OR 0.45, 95% CI 0.20–1.04, *p* = 0.062). Age, sex, antibiotic exposure, and SAPS II were not significant. An mNGS × blood culture interaction model (Figure 7B) showed no evidence that the mNGS effect differed by blood culture status (OR 1.04, 95% CI 0.02–58.34, *p* = 0.982), and the main mNGS association persisted (OR 2.65, 95% CI 1.21–5.80, *p* = 0.016).

## 4. Discussion

### 4.1. Principal Findings

Active antimicrobial treatment adaptation occurred in 49.8% of the 243 analytic episodes within the integrated stewardship pathway. This suggests that plasma mNGS served as a clinically relevant decision-support input alongside blood culture, biomarker kinetics, and bedside assessment. Consistent with this, mNGS positivity was independently associated with greater odds of an active stewardship change (OR 2.66, 95% CI 1.23–5.75, *p* = 0.014). At the patient level, in-hospital survival did not differ significantly between patients in whom empirical therapy was maintained and those undergoing stewardship-guided antimicrobial adaptation (log-rank *p* = 0.951; unadjusted HR 1.02, 95% CI 0.57–1.81).

Together, these findings suggest that mNGS functioned primarily as a clinical decision-support tool during the 2023–2024 study period, complementing conventional microbiology and clinical assessment rather than serving as an independent prognostic marker. As no ICU stewardship guidelines yet existed for integrating plasma mNGS into antimicrobial decision-making, results were interpreted through multidisciplinary clinical judgment alongside microbiology and biomarker kinetics—supporting, rather than replacing, established stewardship principles.

Clinical mNGS has continued to evolve rapidly since the study period. Current platforms increasingly offer resistance-gene profiling [14], longitudinal pathogen-burden monitoring [15], and faster turnaround times [16]. The decision-support value observed here may therefore be a conservative estimate of the technology’s current capability.

### 4.2. Observed Predictor Patterns for Hospital Mortality and Stewardship Decisions

Hospital mortality was predominantly associated with physiologic severity and host-response dynamics: higher SAPS II was associated with mortality from admission onward, while PCT values and PCT change became informative in the subsequent phases, joined by temperature in the later phase—consistent with the established prognostic value of these markers in critical illness [9,17,18,19,20,21]. Neither blood culture positivity nor mNGS positivity was independently associated with mortality at any phase.

A different pattern emerged for antimicrobial decision-making. mNGS positivity was independently associated with active treatment modification, whereas admission severity was not. Blood culture positivity showed an inverse point estimate for treatment modification, and the mNGS × blood culture interaction provided no evidence that mNGS’s association with stewardship action differed by blood-culture status—though this should not be considered an absolute finding because the estimate was imprecise with a wide confidence interval. Together, these patterns indicate that microbiological diagnostics and physiologic severity served distinct clinical roles within the stewardship pathway: microbiological diagnostics informed antimicrobial adaptation, while physiologic severity tracked prognosis.

The survival comparison closes the loop. Patients undergoing stewardship-guided adaptation and those maintained on empirical therapy showed no significant difference in overall in-hospital survival (log-rank *p* = 0.9507; unadjusted HR 1.02, 95% CI 0.57–1.81). Read together with the Phase B/C decision models and the mortality models above, these findings indicate similar observed survival between the two stewardship groups, while mortality remained more closely associated with physiologic severity and biomarker dynamics—particularly SAPS II and PCT—than with pathogen identification itself.

### 4.3. Anaerobic/Gut-Associated Escalation

The 50% escalation rate among anaerobic/gut-associated pathogens is notable in this context. In the same source cohort, we previously showed that mNGS detection remained stable under antibiotic exposure, whereas blood culture yield declined sharply [3]. Similar observations have been reported in other ICU cohorts, where mNGS-exclusive detection of anaerobes such as Bacteroides fragilis and Prevotella prompted antibiotic escalation [22].

### 4.4. Relationship to Previous mNGS Studies

Previous studies reported mNGS-associated antibiotic adjustment across different ICU settings [23,24,25,26,27] (Table 4). Potential therapy modification was identified in 53% [23] and 32.6% [24] of cases by retrospective expert review, while Liu et al. reported microbiology-driven adjustment in 52.5% [25]. Our observed treatment-adaptation rate of 49.8% was broadly comparable despite substantial methodological differences across studies.

A recent ICU study in severe community-acquired pneumonia found that earlier mNGS-guided antibiotic adjustment was independently associated with lower 28-day mortality, emphasizing the potential importance of timely integration of microbiological information rather than diagnostic yield alone. However, the study population and binary timing model differed from ours [4]. Conversely, a recent multicenter ICU cohort found mNGS influenced therapy in only 5.3% of patients, a finding attributed largely to a median turnaround time of 14 days and frequent use of mNGS as a last-resort diagnostic test [5].

Consistent with a continuous-integration approach, our framework combines BC and mNGS findings with clinical and biomarker information rather than interpreting mNGS in isolation [28]. This reflects a cautious strategy given the current limitations of mNGS as a stand-alone diagnostic tool. As turnaround times, interpretive frameworks, and prospective validation improve, the role of mNGS within integrated stewardship pathways may expand [29].

### 4.5. Limitations

This study has several limitations. First, its retrospective, single-center observational design limits generalizability and precludes causal inference. After reconstruction from the original 393 sampling episodes (302 patients) to the documented analytic cohort of 243 eligible episodes, some microbiological and antibiotic-exposure strata became very small. This reflects the unique-patient cohort definition, repeated sampling in some patients, and the predefined exclusions shown in the STROBE flow diagram and Methods section. Second, confounding by indication is possible because empirical antibiotic therapy was initiated based on the overall clinical suspicion of infection, including biomarker findings, suspected diagnosis, and illness severity. Blood cultures and mNGS were obtained within this same clinically driven diagnostic and treatment pathway. Third, regression models were developed and evaluated within the same cohort and have not undergone external validation. Fourth, despite the use of penalized regression and patient-clustered analyses where appropriate, the limited sample size, small number of outcome events, missing biomarker data, and multiple exploratory analyses may increase the risk of unstable estimates and overfitting. These findings should therefore be interpreted within the context of an exploratory observational study. Fifth, plasma mNGS detects microbial cfDNA rather than viable organisms, and despite bioinformatic filtering, reagent- and laboratory-derived background DNA cannot be completely excluded, particularly for low-abundance signals. In addition, detailed run-level quality-control metrics, background-subtraction methods, contaminant-filtering procedures, and reporting thresholds of the DISQVER^®^ pipeline were not available to the investigators, limiting independent assessment and reproducibility of the analytical workflow. Finally, the study did not assess costs or cost-effectiveness.

## 5. Conclusions

In this retrospective ICU cohort, plasma mNGS provided complementary microbiological information that was associated with antimicrobial escalation, de-escalation, continuation, and discontinuation within a structured four-phase decision framework. The framework integrated clinical assessment, biomarker kinetics, and conventional microbiology. Nearly half of the analytic episodes underwent at least one antimicrobial treatment adaptation, while hospital mortality did not differ significantly between patients who received stewardship-guided adaptation and those in whom empirical therapy was maintained.

The factors associated with antimicrobial stewardship decisions differed from those associated with mortality. This suggests that plasma mNGS and blood culture primarily support microbiological and therapeutic decision-making, whereas biomarker dynamics—particularly SAPS II and procalcitonin—were more closely linked to patient prognosis.

Prospective, multicenter, and ideally comparative studies are needed to externally validate the proposed framework, define standardized implementation strategies, and assess its clinical and economic impact.

## Figures and Tables

**Figure 1:**
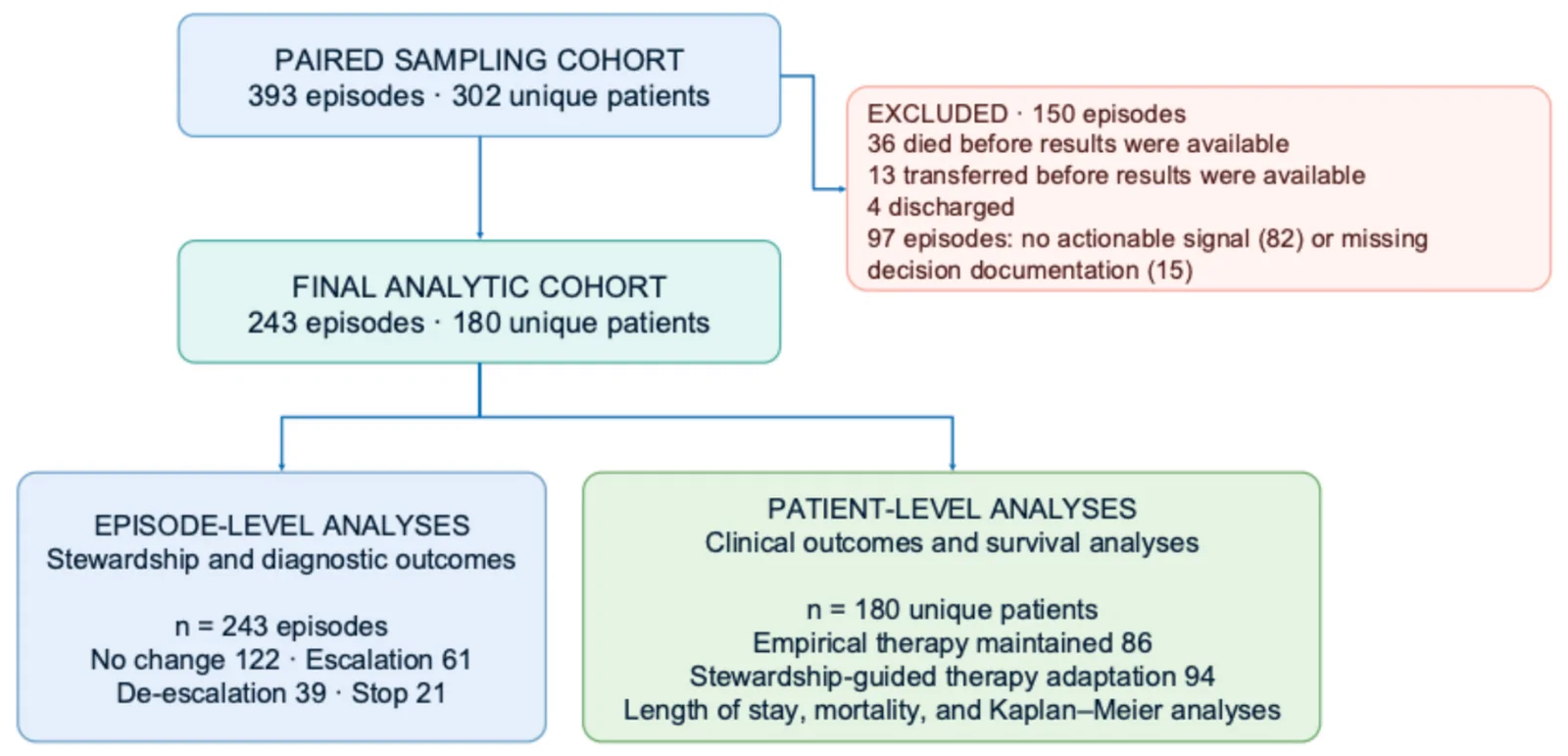
STROBE flow diagram of cohort selection and analysis populations.

**Figure 2:**
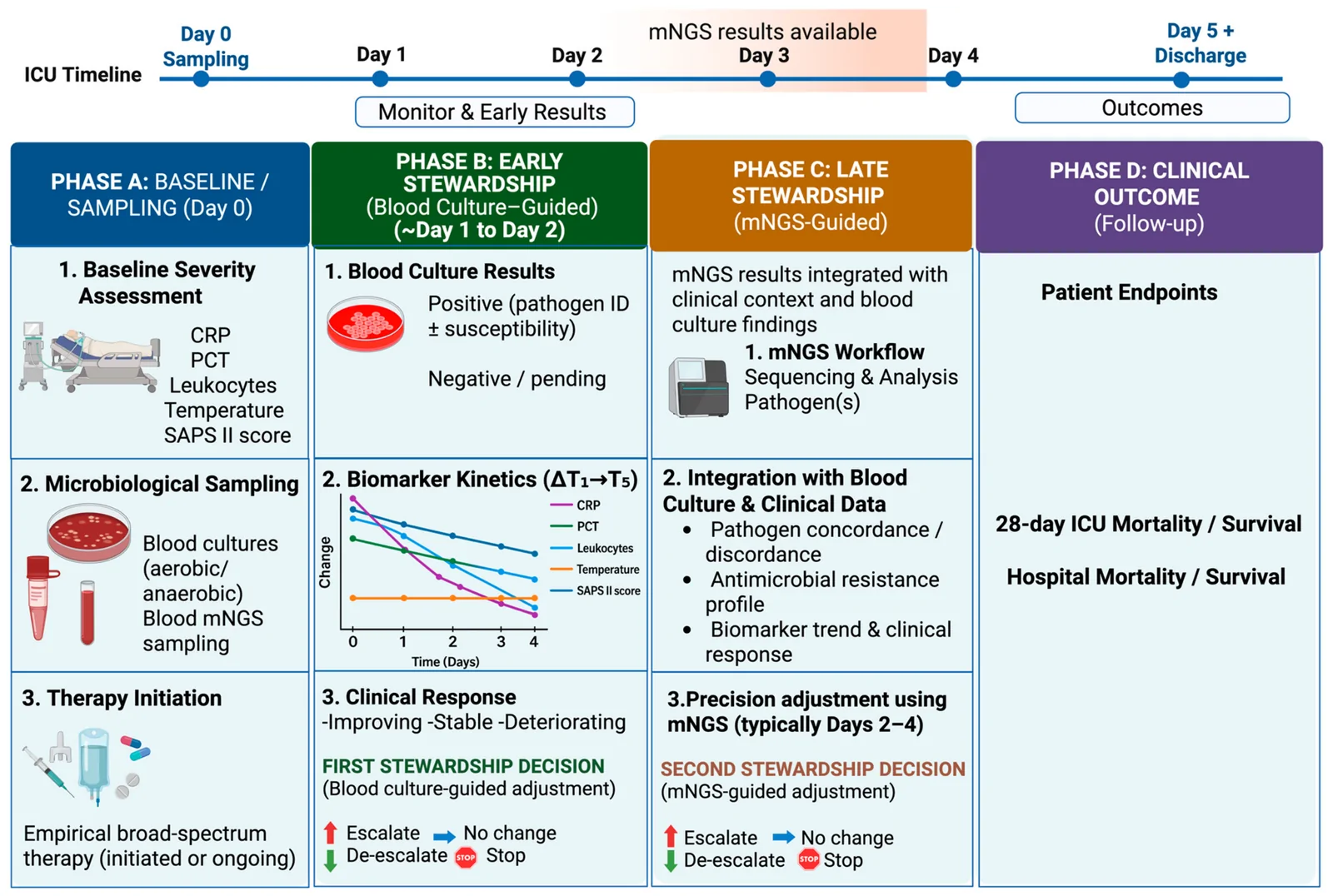
Four-phase mNGS-guided antibiotic stewardship framework. Schematic overview of the ICU decision model integrating admission severity, biomarker kinetics, microbiological findings, stewardship decisions, and clinical outcome during mNGS-guided management. Created in BioRender (https://BioRender.com/rohzlzi accessed on 1 October 2026).

**Figure 3:**
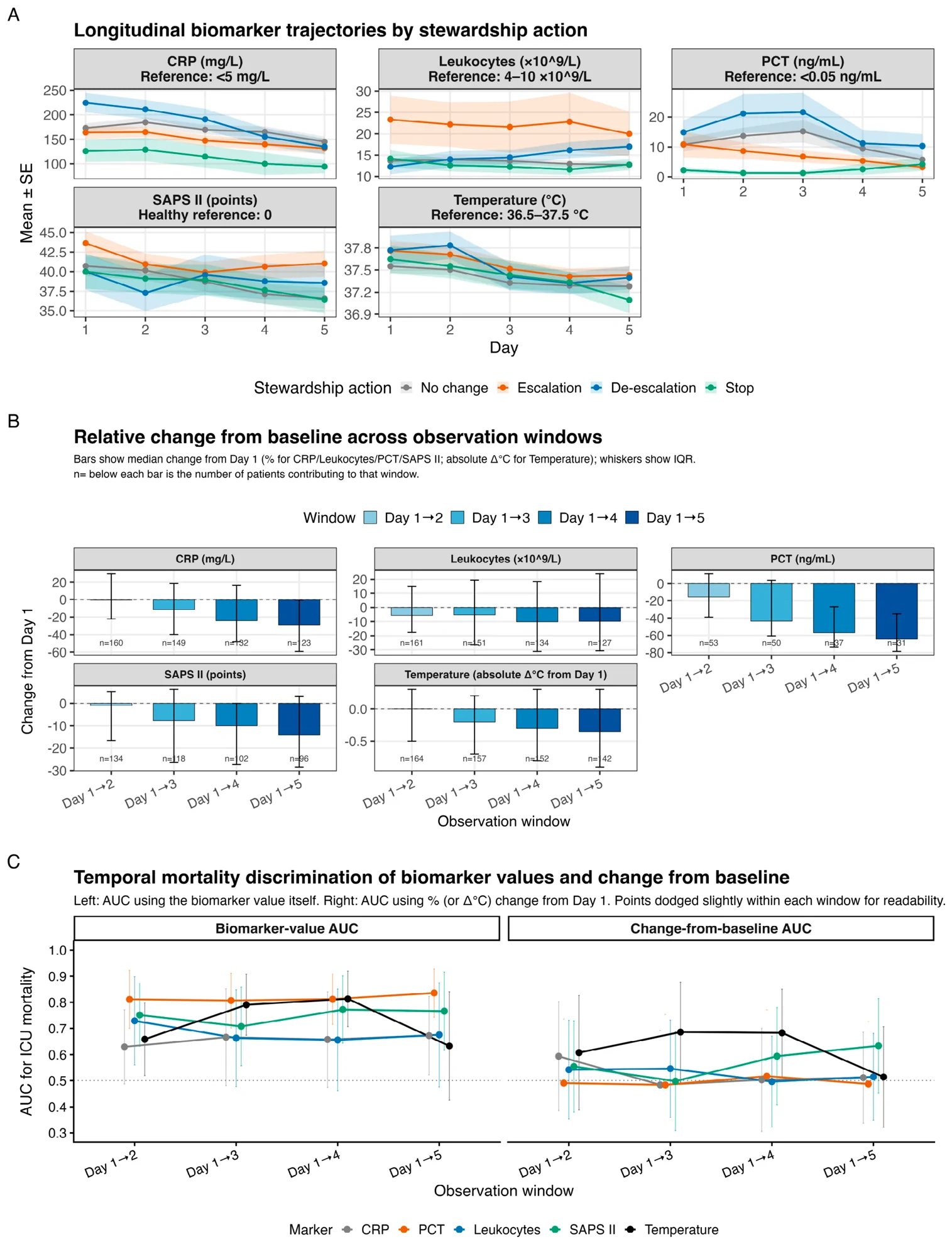
Longitudinal biomarker trajectories, changes from baseline, and temporal discrimination of ICU mortality. (**A**) Daily biomarker values by stewardship action. (**B**) Median changes from Day 1 across observation windows, with interquartile ranges. (**C**) AUCs with 95% confidence intervals for ICU mortality using biomarker values and changes from baseline.

**Figure 4:**
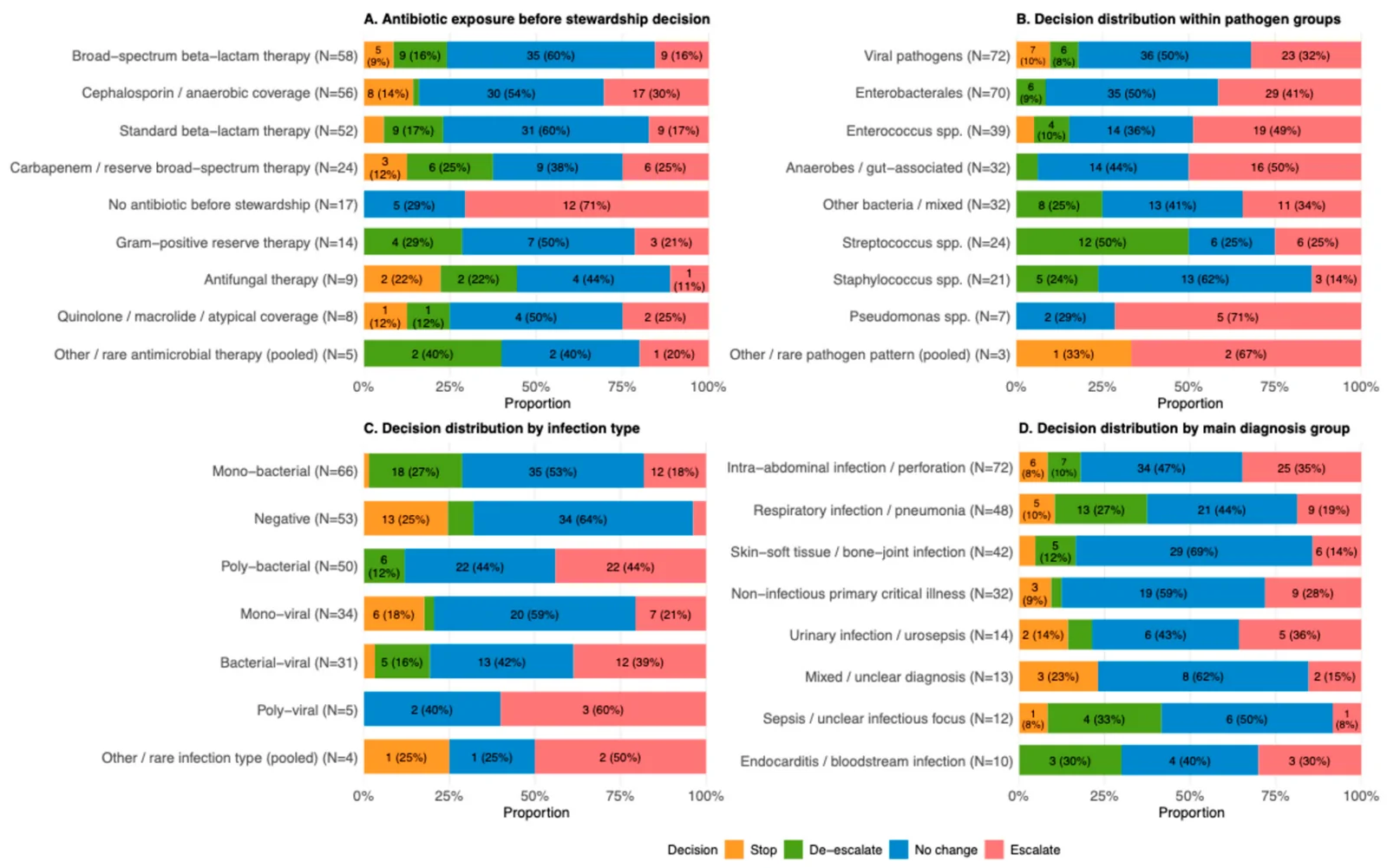
Clinical determinants of ICU antibiotic stewardship decisions. (**A**) Stewardship decisions by antibiotic regimen administered before review. Antimicrobial regimens were classified into clinically defined treatment groups: Standard β-lactam therapy (ampicillin, ampicillin/sulbactam, amoxicillin/clavulanic acid, penicillin G, flucloxacillin, cefazolin, cefuroxime); Cephalosporin/anaerobic coverage (ceftriaxone, cefotaxime, metronidazole); Broad-spectrum β-lactam therapy (piperacillin/tazobactam, cefepime, ceftazidime); Carbapenem/reserve broad-spectrum therapy (meropenem, imipenem); Gram-positive reserve therapy (vancomycin, linezolid); Quinolone/macrolide/atypical coverage (ciprofloxacin, levofloxacin, clarithromycin, roxithromycin); Antifungal therapy (fluconazole, voriconazole, caspofungin, anidulafungin, amphotericin B); No antibiotic before stewardship; and Other/rare antimicrobial therapy (including clindamycin and antiviral agents such as aciclovir, valaciclovir, and ganciclovir). (**B**–**D**) Stewardship decisions stratified by pathogen group, infection type, and primary clinical diagnosis, respectively. Horizontal stacked bars show the proportion of episodes resulting in antibiotic discontinuation (Stop), de-escalation, continuation without change (No change), or escalation. Numbers in parentheses indicate the number of episodes in each category, and percentages represent within-group (row) proportions. The final analytic cohort comprised 243 ICU episodes.

**Figure 5:**
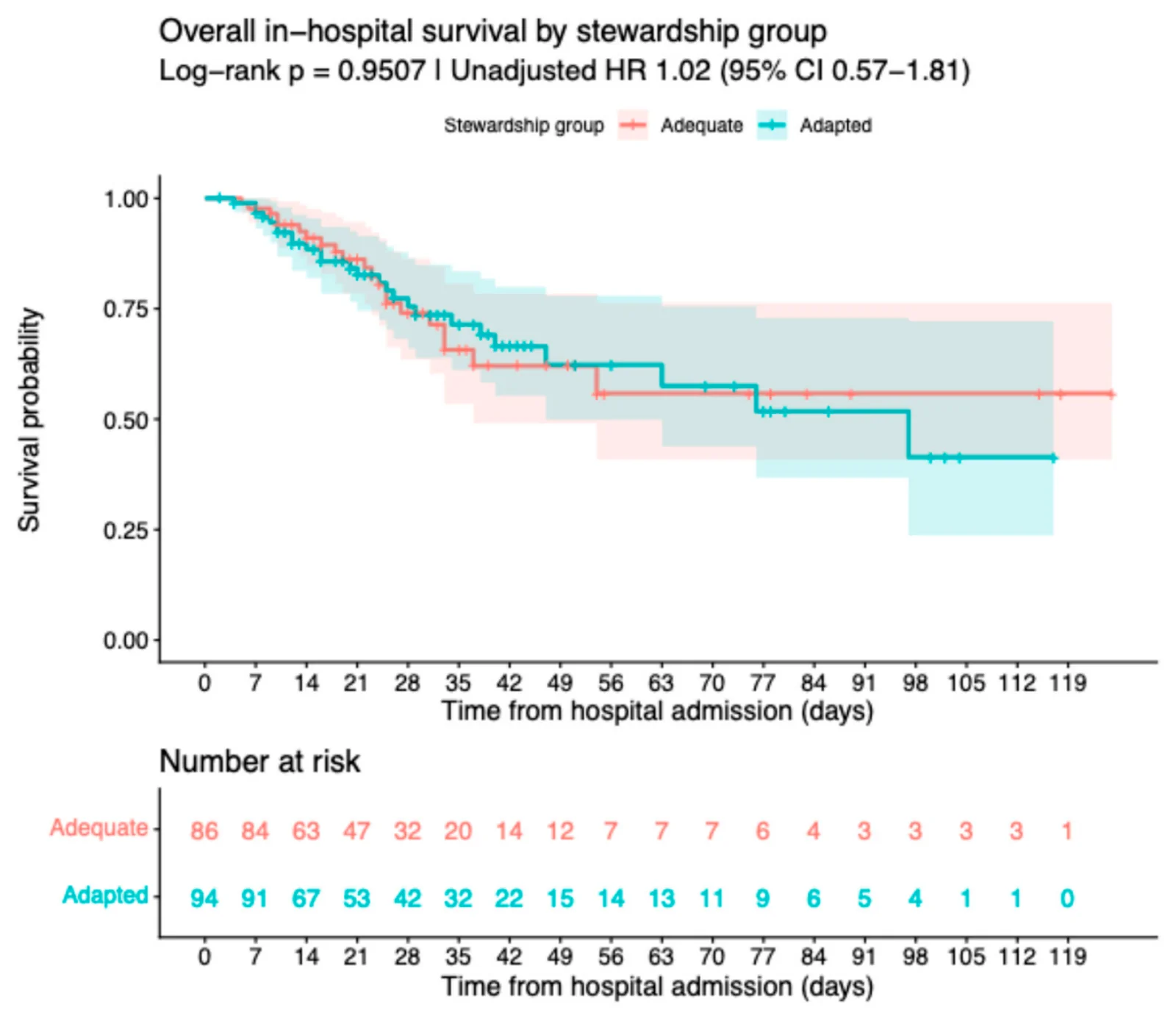
Overall in-hospital survival according to antimicrobial stewardship strategy. Kaplan–Meier curves compare patients in whom empirical antimicrobial therapy was maintained with those undergoing stewardship-guided antimicrobial adaptation. Shaded areas indicate 95% confidence intervals. The between-group comparison was assessed using the log-rank test, and the unadjusted hazard ratio was estimated using Cox proportional-hazards regression.

**Figure 6:**
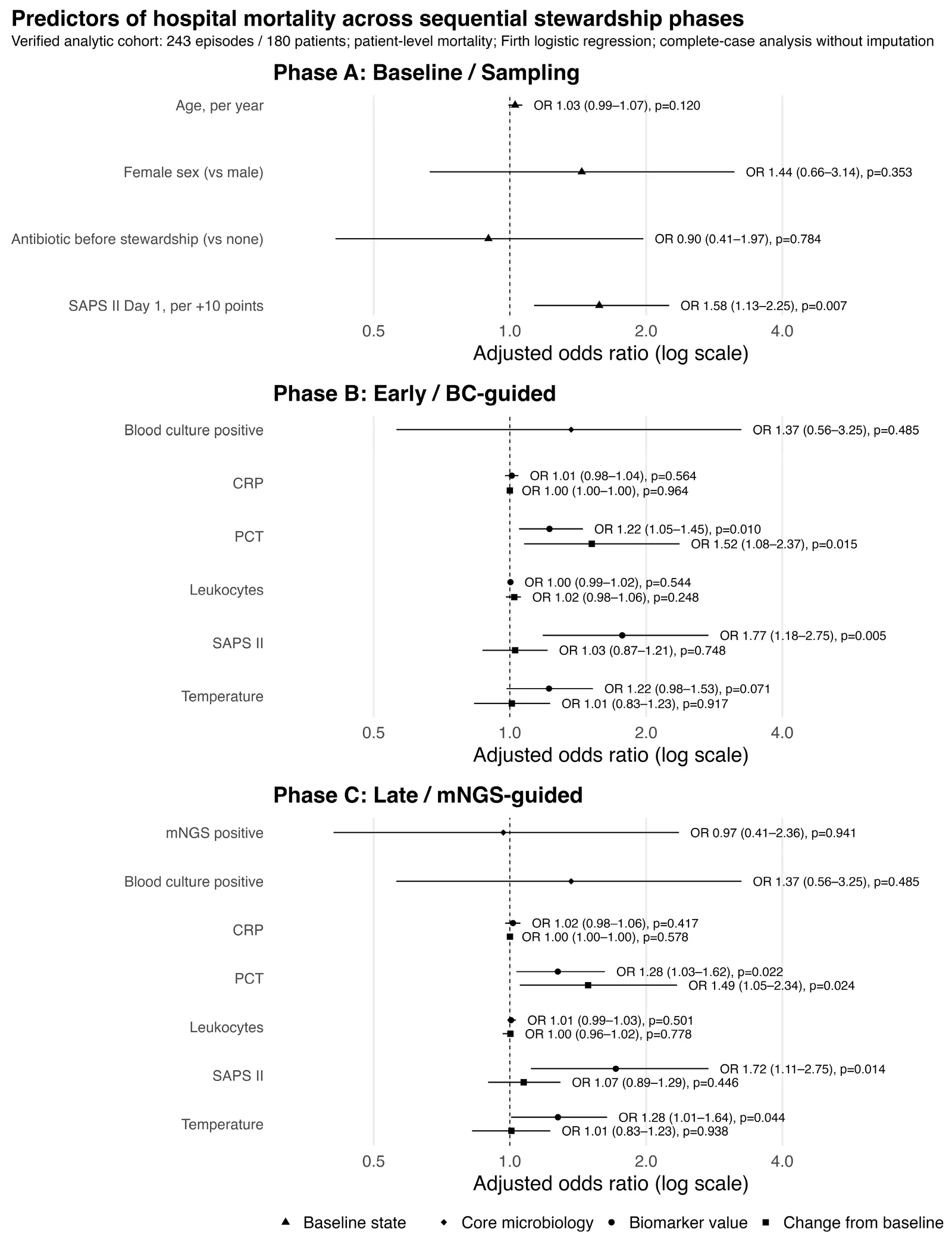
Sequential predictors of hospital mortality. Patient-level Firth logistic regression models across admission (**Phase A**), early blood-culture-guided (**Phase B**), and late mNGS-guided (**Phase C**) phases. Adjusted ORs with 95% CIs are shown. Absolute biomarker values and change-from-baseline measures were fitted in separate models.

**Figure 7:**
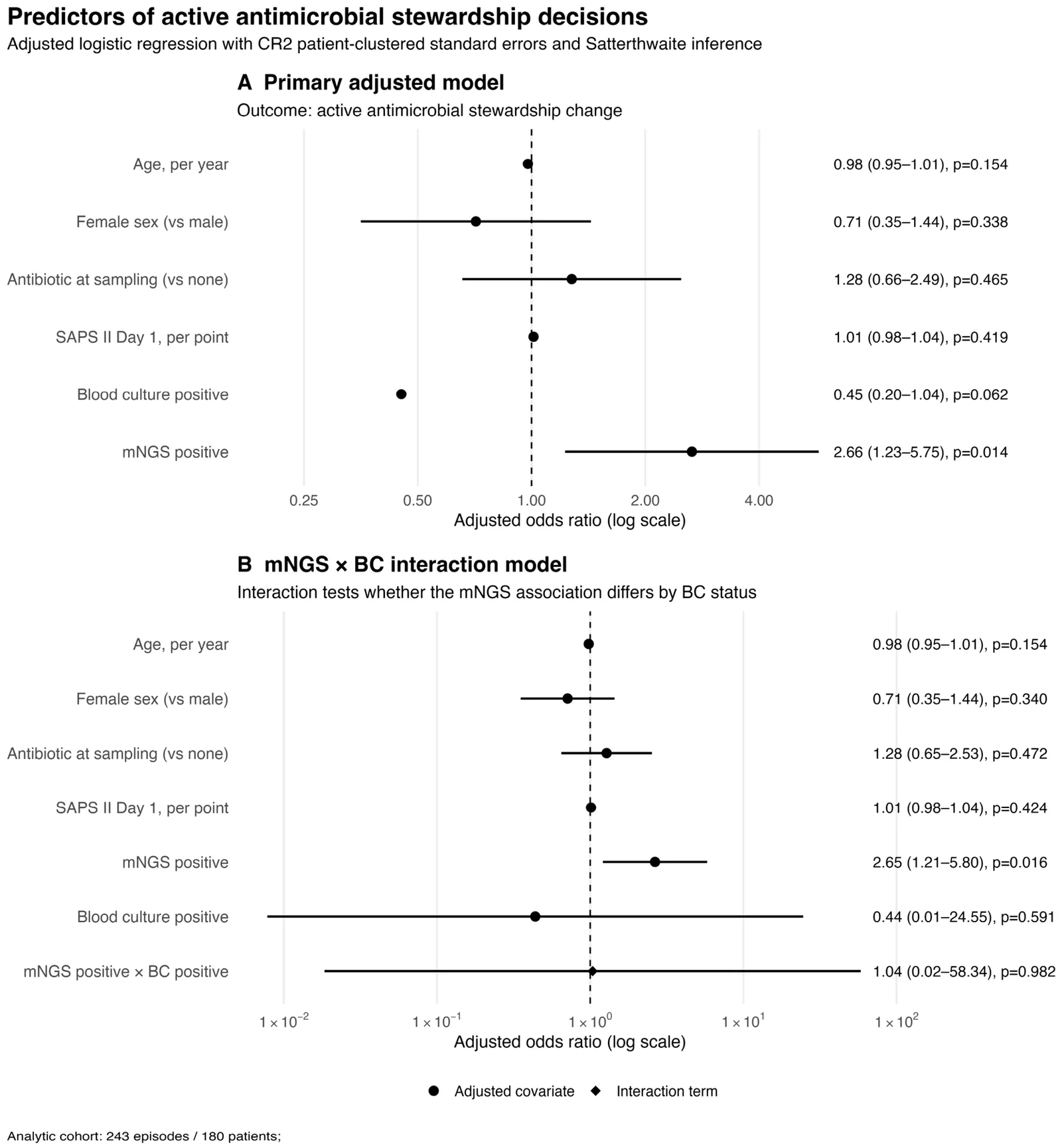
Predictors of active antimicrobial stewardship decisions. Adjusted logistic regression models for active treatment modification (escalation, de-escalation, or discontinuation vs. no change) across 243 episodes from 180 patients. Panel (**A**) shows the primary model; Panel (**B**) includes the mNGS × blood culture interaction. ORs with 95% CIs are shown; inference used CR2 patient-clustered standard errors with Satterthwaite correction.

**Table 1:** (**A**). Baseline demographic and comorbidity characteristics of unique patients according to subsequent stewardship status. Values are median [Q1, Q3] or *n* (%). *p*-values: Wilcoxon rank-sum test for age and Fisher’s exact test for categorical variables. SMD = absolute standardized mean difference. (**B**). Day 0 severity and laboratory characteristics of analytic sampling episodes according to episode-level stewardship status. Values are median [Q1, Q3]. *p*-values: Wilcoxon rank-sum test. SMD = absolute standardized mean difference.

(**A**)							
**Characteristic**	***n***	**Overall** **(*n* = 180)**		**Empirical Therapy** **Maintained (*n* = 86)**	**Stewardship-Guided** **Therapy Adaptation (*n* = 94)**	***p*** **-Value**	**SMD**
Age, years	180	73.0 [64.5, 81.0]		74.0 [67.0, 82.0]	72.5 [61.0, 81.0]	0.166	0.26
Sex	180					0.274	0.18
Male		117 (65.0%)		52 (60.5%)	65 (69.1%)		
Female		63 (35.0%)		34 (39.5%)	29 (30.9%)		
Arterial hypertension	176	95 (54.0%)		49 (59.0%)	46 (49.5%)	0.227	0.19
Cardiovascular disease	177	117 (66.1%)		60 (71.4%)	57 (61.3%)	0.203	0.22
Pulmonary disease	177	80 (45.2%)		37 (44.0%)	43 (46.2%)	0.880	0.04
Renal disease	178	72 (40.4%)		34 (40.0%)	38 (40.9%)	>0.9	0.02
History of stroke	178	17 (9.6%)		10 (11.8%)	7 (7.5%)	0.445	0.14
Diabetes mellitus	177	64 (36.2%)		35 (41.2%)	29 (31.5%)	0.211	0.20
(**B**)							
**Characteristic** **(Day 0)**	***n***	**Missing**	**Overall (*n* = 243 episodes)**	**No active stewardship adaptation (*n* = 122)**	**Active stewardship adaptation (*n* = 121)**	***p*** **-value**	**SMD**
SAPS II	205	38	41.0 [34.0, 49.0]	41.0 [32.5, 50.0]	42.0 [35.0, 48.0]	>0.9	0.02
CRP, mg/L	234	9	159.6 [92.7, 253.0]	157.9 [86.9, 253.0]	159.6 [105.1, 252.2]	0.478	0.13
PCT, ng/mL	153	90	1.1 [0.3, 11.0]	1.1 [0.3, 13.5]	1.1 [0.4, 6.7]	0.882	0.09
Leukocytes, /nL	236	7	12.1 [8.3, 17.9]	11.6 [8.2, 17.8]	12.7 [8.5, 18.9]	0.471	0.22
Temperature, °C	222	21	37.5 [36.9, 38.3]	37.3 [36.8, 38.2]	37.6 [36.9, 38.4]	0.118	0.19
Creatinine, mg/dL	227	16	1.2 [0.8, 2.1]	1.2 [0.8, 2.0]	1.3 [0.8, 2.2]	0.713	0.06

**Table 2:** Clinical outcomes according to patient-level stewardship status. Values are median [Q1, Q3] or n (%). *p*-values: Wilcoxon rank-sum test for length-of-stay outcomes and Fisher’s exact test for mortality outcomes. No SMD is reported for post-baseline outcomes.

Outcome	*n*	Overall (*n* = 180)	Empirical Therapy Maintained (*n* = 86)	Stewardship-Guided Therapy Adaptation (*n* = 94)	*p*-Value	SMD
ICU length of stay, days	180	9.0 [4.0, 19.0]	7.0 [4.0, 15.0]	12.0 [6.0, 24.0]	0.003	0.46
Hospital length of stay, days	180	23.0 [12.0, 38.0]	23.0 [12.0, 33.0]	23.5 [12.0, 40.0]	0.480	0.11
ICU mortality	180				0.487	0.11
Survived		160 (88.9%)	78 (90.7%)	82 (87.2%)		
Died		20 (11.1%)	8 (9.3%)	12 (12.8%)		
Hospital mortality	180				0.734	0.07
Survived		133 (73.9%)	65 (75.6%)	68 (72.3%)		
Died		47 (26.1%)	21 (24.4%)	26 (27.7%)		

**Table 3:** Antibiotic treatment adaptations according to mNGS, blood culture, and antibiotic exposure. Stratum counts are based on the 243 analytic sampling episodes derived from 180 unique ICU patients. Treatment adaptations were categorized as no change, escalation, de-escalation, or discontinuation of antibiotic therapy.

mNGS	BC	Abx	Episodes (*n*)	No Change	Escalation	De-Escalation	Stop	Total Adaptations *n* (%)
+	-	+	92	39 (42.4%)	27 (29.3%)	17 (18.5%)	9 (9.8%)	53 (57.6%)
+	+	+	11	5 (45.5%)	1 (9.1%)	5 (45.5%)	0 (0.0%)	6 (54.5%)
+	-	-	50	20 (40.0%)	23 (46.0%)	7 (14.0%)	0 (0.0%)	30 (60.0%)
+	+	-	38	23 (60.5%)	9 (23.7%)	6 (15.8%)	0 (0.0%)	15 (39.5%)
-	-	+	46	30 (65.2%)	1 (2.2%)	3 (6.5%)	12 (26.1%)	16 (34.8%)
-	+	+	4	3 (75.0%)	0 (0.0%)	1 (25.0%)	0 (0.0%)	1 (25.0%)
-	-	-	2	2 (100.0%)	0 (0.0%)	0 (0.0%)	0 (0.0%)	0 (0.0%)
Total			243	122 (50.2%)	61 (25.1%)	39 (16.0%)	21 (8.6%)	121 (49.8%)

**Table 4:** Comparison of the current study with selected clinical mNGS studies in critically ill patients. Studies are summarized according to population and setting, sample size, sequencing platform and specimen type, turnaround time, diagnostic yield relative to conventional microbiology, reported antimicrobial treatment modification, and clinical outcomes. Abbreviations: Abx, antibiotics; BC, blood culture; BSI, bloodstream infection; CMT, conventional microbiological testing; LRTI, lower respiratory tract infection; mcfDNA, microbial cell-free DNA; mNGS, metagenomic next-generation sequencing; TAT, turnaround time.

Study	Population/Setting	*n*	Platform/Sample	TAT	mNGS vs. Comparator	Treatment Change	Outcome
Current study	Adult ICU; paired plasma mNGS-BC; single-center, retrospective	180 patients 243 episodes	DISQVER/Noscendo; plasma cfDNA	~48–96 h	Bacterial mNGS 51–53%; BC 30.4% (Abx−) vs. 10.4% (Abx+)	49.8% treatment adaptation	Hospital mortality 26.1%; no significant difference between adequate and adjusted antibiotic therapy.
Grumaz et al., 2019 [23]	Surgical ICU; septic shock; prospective observational, single-center	48 patients 256 plasma samples	Plasma cfDNA NGS with relevance scoring	~24 h	At sepsis onset: 72% vs. 33% BC; overall: 71% vs. 11% BC	53% potential therapy modification, retrospectively assessed by expert panel	28-day mortality 19%; 90-day mortality 32%; higher 28- and 90-day survival in patients retrospectively classified as receiving adequate antimicrobial therapy.
Brenner et al., 2025 [24]	ICU/Sepsis/septic shock; prospective observational, multicenter	491 samples	Plasma mcfDNA NGS; SIQ-based analysis	Retrospective mNGS analysis	70.5% vs. 19.4% BC	32.6% treatment adaptation	28-day mortality 26.7%; no significant difference between adequate and adjusted antibiotic therapy.
Liu et al., 2023 [25]	ICU; suspected BSI/sepsis; retrospective	162 cases	Plasma DNA mNGS; Illumina NextSeq 550	Not clearly reported	68.5% vs. 26.5% BC; 47.5% vs. 26.5% excluding viruses	Microbiology-driven antibiotic adjustment: 52.5% (85/162)	No reported difference in mortality or LOS between antibiotic-adjustment groups.
Xu et al., 2026 [26]	ICU; suspected LRTI; mixed specimens	261 patients	DIFSEQ-200 DNA/RNA mNGS	~24 h	55.6% both positive; 19.9% mNGS-only; 13.8% CMT-only	mNGS-guided treatment change: 49.0% (128/261)	In-hospital mortality 24.1%; no comparative mortality analysis by treatment-adjustment group.
Kling et al., 2024 [27]	Critically ill; suspected pneumonia; BAL, single-center, retrospective	47 patients	RPIP targeted NGS; MiSeq; BAL, BioFire^®^ FilmArray^®^ Pneumonia Panel	Not reported	Sensitivity 63.9%; specificity 98.1% vs. culture	17/38 (44.5%) potential antibiotic addition	No demonstrated mortality association

## Data Availability

The data presented in this study are available from the corresponding author upon reasonable request. Due to ethical and data-protection requirements, original patient-level data that could permit identification or tracking of individual patients cannot be shared outside the hospital.

## Supplementary Materials

The following supporting information can be downloaded at: https://www.mdpi.com/article/10.3390/antibiotics15100978/s1, Figure S1. Read-count distributions across microbial domains; Figure S2. Bacterial mNGS read-count distributions by species; Figure S3. Viral mNGS read-count distributions by species; Figure S4. Fungal mNGS read-count distributions by species; Figure S5. Correlation of domain-level mNGS read burden with baseline host-response markers; Figure S6. Correlation of bacterial genus-level read burden with base-line host-response markers; Table S1. Phase-specific sensitivity analyses for ICU mortality using Firth penalized logistic regression; Table S2. Patient-level mortality in episodes without an evaluable stewardship decision versus the analytic cohort; Table S3. Detailed antimicrobial treatment adaptations ac-cording to mNGS and blood-culture findings.
