# Supplementary material for: Integrating Plasma mNGS into ICU Antimicrobial Stewardship: A Four-Phase Framework
Source: Antibiotics (Basel). 2026 Oct 3;15(10):978. doi: 10.3390/antibiotics15100978 (PMC13700567; doi:10.3390/antibiotics15100978)
Supplement: Supplementary file 1 [file antibiotics-15-00978-s001.zip › antibiotics-4528574-Supplementary S1.pdf]

## Supplementary Material S1

# Integrating Plasma mNGS into ICU Antimicrobial Stewardship: A Four-Phase Framework

**Torsten H. Schroeder M.D., Ph.D., Mamoun Eliwi Alsaffan M.D., Angelina Bondarenko and Albion Dervishi M.D.**

medius KLINIK Nürtingen, Academic Teaching Hospital of the University Hospital, Tübingen, Departments of Anesthesiology and Intensive Care Medicine, Internal Medicine, and Emergency Medicine

* Correspondence: Author: Torsten H. Schroeder, M.D., Ph.D. Department of Anesthesiology and Intensive Care Medicine. Auf dem Säer 1, 72622 Nürtingen, Germany; torsten.schroeder@uni-tuebingen.de, phone +49 7022 78-21200. Academic Teaching Hospital of the University Hospital, Tübingen.

### Plasma mNGS Read-Count Distributions and Correlation with Baseline Host-Response Markers

This supplement summarizes the plasma mNGS results reported by Noscendo GmbH (DISQVER) for the study cohort of 133 samples out of the 243 cohort. Vendor-reported reads were stratified by microbial domain (bacteria, fungi, and viruses) and by taxonomic assignment. Effective scaled read counts were subsequently correlated with baseline (Day 0) host-response markers, including C-reactive protein (CRP), leukocyte count, procalcitonin (PCT), SAPS II score, and temperature, using Spearman rank correlation. Figures S1–S6 summarize the distribution of detected reads and the corresponding exploratory correlations at the microbial-domain, taxon, and bacterial-genus levels.

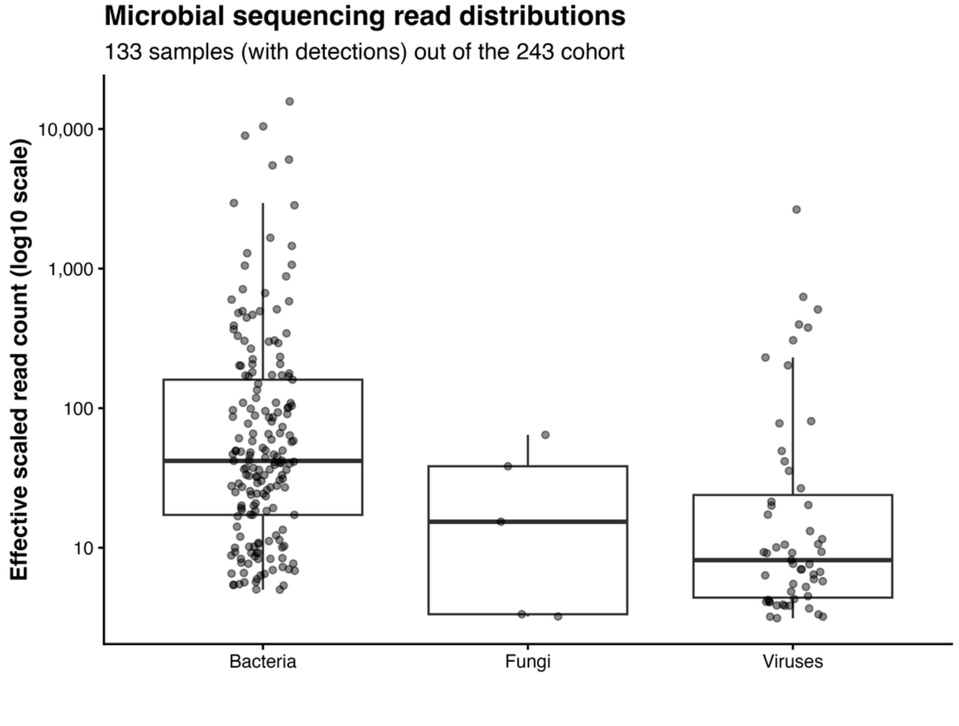

**Figure S1.** Read-count distributions across microbial domains. Effective scaled plasma mNGS read counts (log10 scale) for bacteria, fungi, and viruses across the verified manuscript cohort (n = 133 samples). Each point represents one sample; boxes show median and interquartile range.

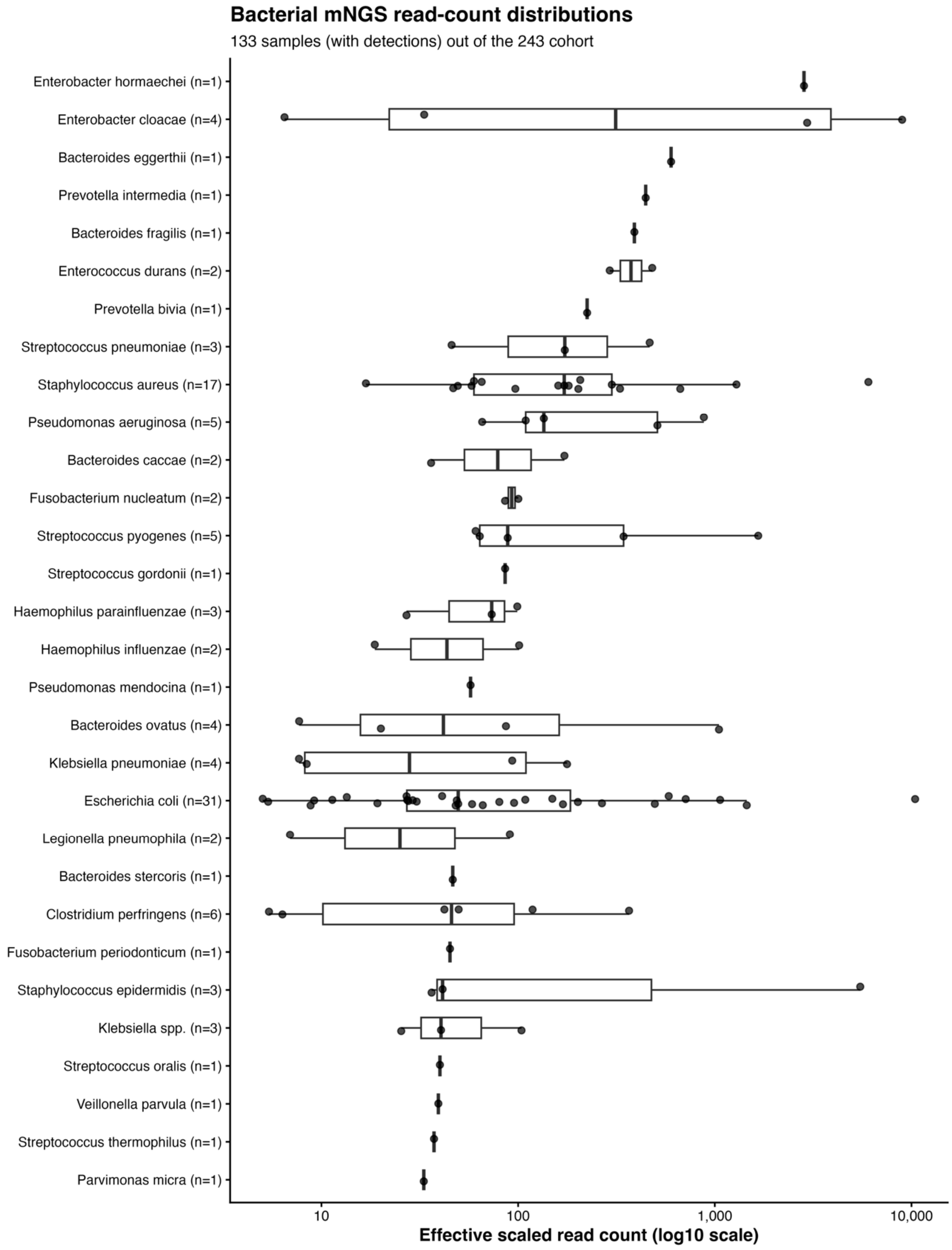

**Figure S2.** Bacterial mNGS read-count distributions by species. Effective scaled read counts (log10 scale) for individual bacterial taxa detected across the cohort (n = 133 samples), ordered by taxon; sample size per taxon is given in parentheses.

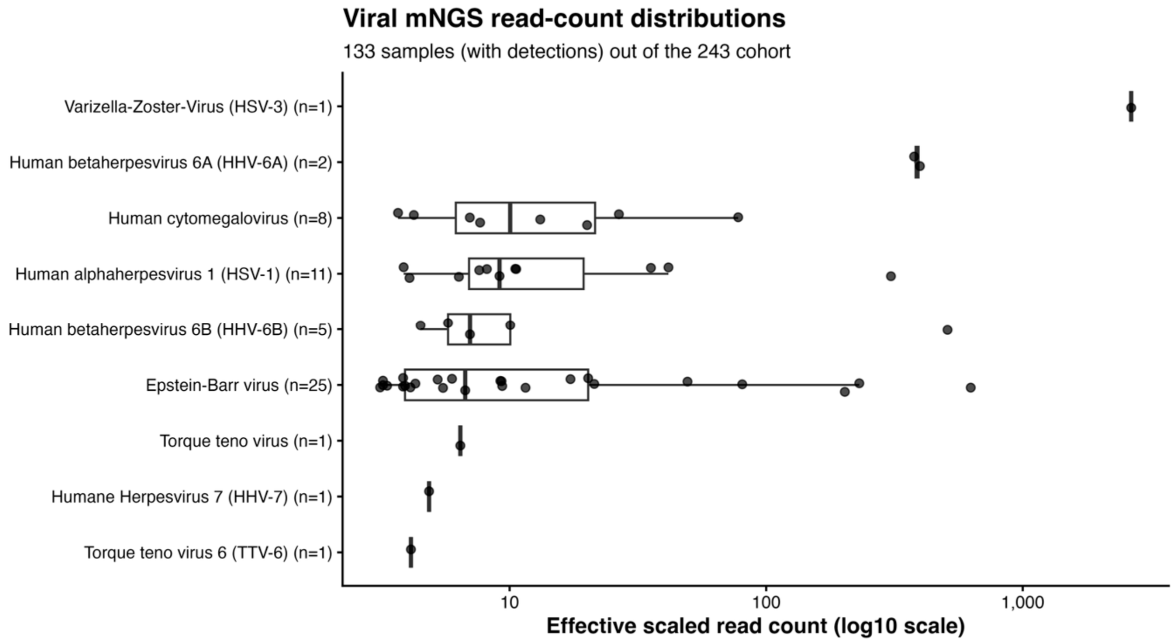


**Figure S3.** Viral mNGS read-count distributions by species. Effective scaled read counts (log10 scale) for individual viral taxa detected across the cohort (n = 133 samples), ordered by taxon; sample size per taxon is given in parentheses.

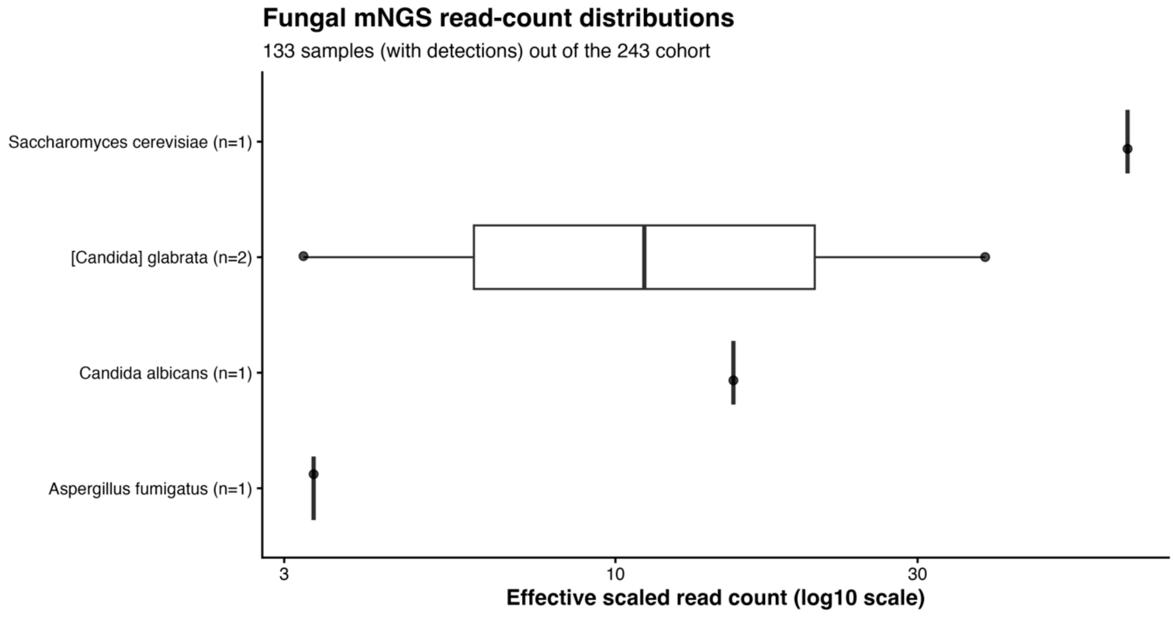

**Figure S4.** Fungal mNGS read-count distributions by species. Effective scaled read counts (log10 scale) for individual fungal taxa detected across the cohort (n = 133 samples), ordered by taxon; sample size per taxon is given in parentheses.

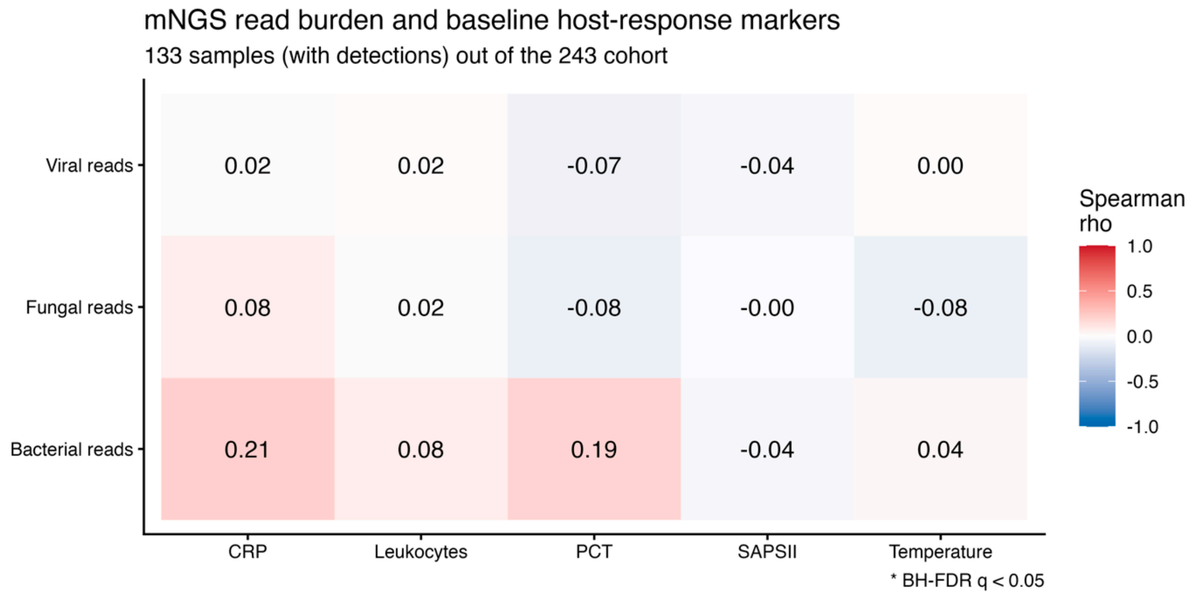


**Figure S5.** Correlation of domain-level mNGS read burden with baseline host-response markers. Spearman correlation coefficients (rho) between effective scaled read counts (bacterial, fungal, viral) and baseline (Day 0) CRP, leukocyte count, procalcitonin (PCT), SAPS II score, and temperature across the whole verified cohort (n = 133). Color scale indicates correlation direction and strength.

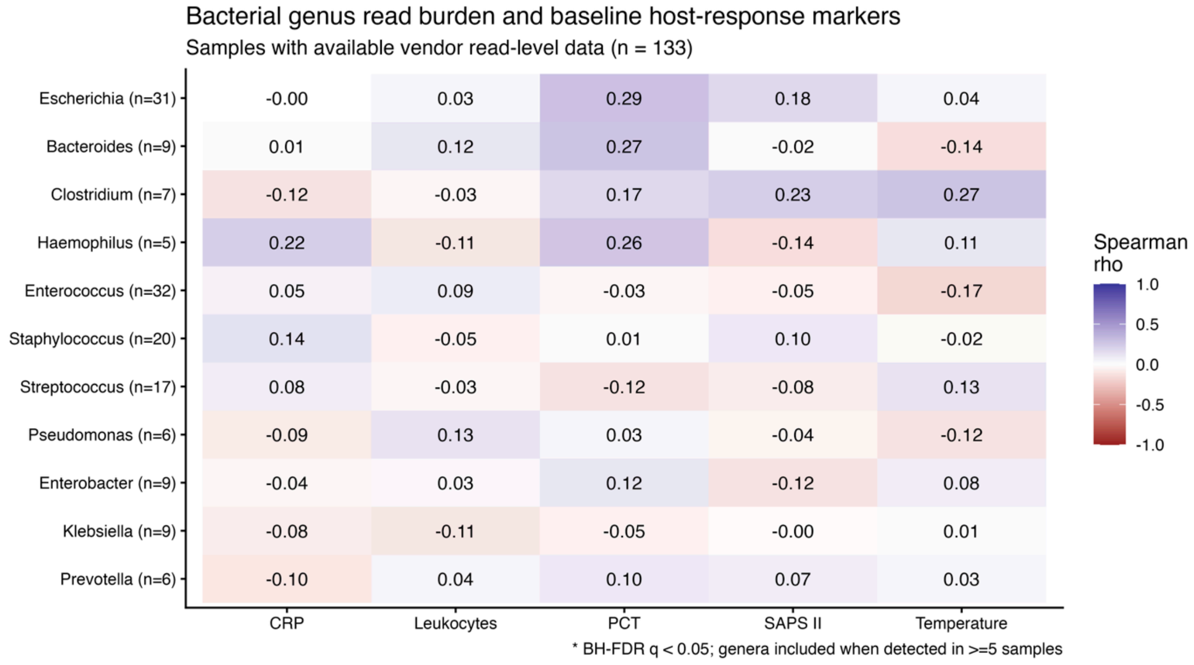


**Figure S6.** Correlation of bacterial genus-level read burden with baseline host-response markers. Spearman correlation coefficients (rho) between effective scaled bacterial read counts, by genus, and baseline (Day 0) CRP, leukocyte count, procalcitonin (PCT), SAPS II score, and temperature. Genera included when detected in ≥ 5 samples across the whole verified cohort.

| N | Deaths | Phase | Model Family | Marker | Outcome | Phase Label | OR_CI | p Value |
|---|---|---|---|---|---|---|---|---|
| 144 | 20 | A | Baseline state | Age, per year | ICU mortality | Phase A: Baseline / Sampling | 1.00 (0.96–1.05) | 0.908 |
| 144 | 20 | A | Baseline state | Female sex (vs male) | ICU mortality | Phase A: Baseline / Sampling | 0.76 (0.26–2.09) | 0.605 |
| 144 | 20 | A | Baseline state | Antibiotic before stewardship (vs none) | ICU mortality | Phase A: Baseline / Sampling | 0.89 (0.33–2.44) | 0.816 |
| 144 | 20 | A | Baseline state | SAPS II Day 1, per +10 points | ICU mortality | Phase A: Baseline / Sampling | 1.74 (1.14–2.76) | **0.010** |
| 179 | 20 | B | Core microbiology | Blood culture positive | ICU mortality | Phase B: Early / BC-guided | 2.23 (0.73–6.63) | 0.157 |
| 177 | 20 | B | Biomarker value | CRP | ICU mortality | Phase B: Early / BC-guided | 1.04 (0.99–1.08) | 0.114 |
| 124 | 20 | B | Biomarker value | PCT | ICU mortality | Phase B: Early / BC-guided | 1.48 (1.19–1.92) | **<0.001** |
| 176 | 20 | B | Biomarker value | Leukocytes | ICU mortality | Phase B: Early / BC-guided | 1.01 (0.99–1.02) | 0.249 |
| 136 | 20 | B | Biomarker value | SAPS II | ICU mortality | Phase B: Early / BC-guided | 1.76 (1.08–2.95) | **0.023** |
| 173 | 18 | B | Biomarker value | Temperature | ICU mortality | Phase B: Early / BC-guided | 1.66 (1.23–2.30) | **<0.001** |
| 168 | 20 | B | Change from baseline | CRP | ICU mortality | Phase B: Early / BC-guided | 1.00 (1.00–1.00) | 0.326 |
| 74 | 13 | B | Change from baseline | PCT | ICU mortality | Phase B: Early / BC-guided | 1.38 (1.02–2.02) | **0.039** |
| 169 | 20 | B | Change from baseline | Leukocytes | ICU mortality | Phase B: Early / BC-guided | 1.03 (0.98–1.07) | 0.236 |
| 134 | 20 | B | Change from baseline | SAPS II | ICU mortality | Phase B: Early / BC-guided | 0.99 (0.80–1.22) | 0.931 |
| 166 | 18 | B | Change from baseline | Temperature | ICU mortality | Phase B: Early / BC-guided | 1.32 (1.02–1.74) | **0.035** |
| 179 | 20 | C | Core microbiology | Blood culture positive | ICU mortality | Phase C: Late / mNGS-guided | 2.04 (0.66–6.19) | 0.208 |
| 179 | 20 | C | Core microbiology | mNGS positive | ICU mortality | Phase C: Late / mNGS-guided | 16.45 (2.05–2131.74) | **0.004** |
| 155 | 20 | C | Biomarker value | CRP | ICU mortality | Phase C: Late / mNGS-guided | 1.04 (0.99–1.09) | 0.153 |
| 86 | 15 | C | Biomarker value | PCT | ICU mortality | Phase C: Late / mNGS-guided | 1.83 (1.29–2.95) | **<0.001** |
| 155 | 20 | C | Biomarker value | Leukocytes | ICU mortality | Phase C: Late / mNGS-guided | 1.01 (0.99–1.04) | 0.215 |
| 103 | 18 | C | Biomarker value | SAPS II | ICU mortality | Phase C: Late / mNGS-guided | 2.68 (1.49–5.40) | **<0.001** |
| 160 | 15 | C | Biomarker value | Temperature | ICU mortality | Phase C: Late / mNGS-guided | 1.57 (1.13–2.30) | **0.007** |
| 149 | 20 | C | Change from baseline | CRP | ICU mortality | Phase C: Late / mNGS-guided | 1.00 (1.00–1.00) | 0.146 |
| 52 | 9 | C | Change from baseline | PCT | ICU mortality | Phase C: Late / mNGS-guided | 1.54 (1.02–2.55) | **0.038** |
| 151 | 20 | C | Change from baseline | Leukocytes | ICU mortality | Phase C: Late / mNGS-guided | 1.00 (0.95–1.02) | 0.771 |
| 102 | 18 | C | Change from baseline | SAPS II | ICU mortality | Phase C: Late / mNGS-guided | 1.21 (0.96–1.55) | 0.110 |
| 154 | 15 | C | Change from baseline | Temperature | ICU mortality | Phase C: Late / mNGS-guided | 1.27 (0.97–1.69) | 0.079 |

**Supplementary Table S1. Phase-specific sensitivity analyses for ICU mortality using Firth penalized logistic regression.** Odds ratios (ORs) with 95% confidence intervals (CIs) are shown for baseline characteristics, microbiological findings, biomarker values, and changes from baseline according to the four-phase stewardship framework. Phase A represents baseline/sampling (Day 0), Phase B early blood culture (BC)-guided stewardship, and Phase C late metagenomic next-generation sequencing (mNGS)-guided stewardship. *N* indicates the number of observations included in each analysis and *Deaths* the number of ICU deaths. Reported *p* values are two-sided. Abbreviations: BC, blood culture; CRP, C-reactive protein; PCT, procalcitonin; SAPS II, Simplified Acute Physiology Score II; mNGS, metagenomic next-generation sequencing; OR, odds ratio; CI, confidence interval.

| Group (patients) | Hospital mortality | p vs 180 (hospital) | ICU mortality | p vs 180 (ICU) |
|---|---|---|---|---|
| Analytic cohort | 47/180 (26.1%) | — | 20/180 (11.1%) | — |
| All 97 excluded episodes | 24/74 (32.4%) | 0.36 | 13/74 (17.6%) | 0.22 |
| No-signal (82) | 18/62 (29.0%) | 0.74 | 8/62 (12.9%) | 0.65 |
| BC+/mNGS- undocumented (15) | 6/12 (50.0%) | 0.09 | 5/12 (41.7%) | 0.01 |

**Supplementary Table S2.** Patient-level mortality in episodes without an evaluable stewardship decision versus the analytic cohort. Excluded groups include only patients with no analytic episode; 17 patients with both types of episode are counted in the analytic cohort. No-signal: mNGS−/BC−/Abx− (82 episodes, 62 patients). Undocumented: BC+/mNGS−/Abx− without a documented decision (15 episodes, 12 patients; 9 likely contaminants, 6 potential pathogens).
