# Supplementary material for: Integrating Plasma mNGS into ICU Antimicrobial Stewardship: A Four-Phase Framework
Source: Antibiotics (Basel). 2026 Oct 3;15(10):978. doi: 10.3390/antibiotics15100978 (PMC13700567; doi:10.3390/antibiotics15100978)
Supplement: Supplementary file 1 [file antibiotics-15-00978-s001.zip › antibiotics-4528574-Supplementary S2.pdf]

Supplementary Material S2

Integrating Plasma mNGS into ICU Antimicrobial Stewardship: A Four-Phase Framework

Torsten H. Schroeder M.D., Ph.D., Mamoun Eliwi Alsaffan M.D., Angelina Bondarenko and Albion Dervishi M.D.

medius KLINIK Nürtingen, Academic Teaching Hospital of the University Hospital, Tübingen, Departments of Anesthesiology and Intensive Care Medicine, Internal Medicine, and Emergency Medicine

* Correspondence: Author: Torsten H. Schroeder, M.D., Ph.D. Department of Anesthesiology and Intensive Care Medicine. Auf dem Säer 1, 72622 Nürtingen, Germany; torsten.schroeder@uni-tuebingen.de, phone +49 7022 78-21200. Academic Teaching Hospital of the University Hospital, Tübingen.

Detailed Episode-Level Antimicrobial Treatment Adaptations

Supplementary Table S3. Detailed antimicrobial treatment adaptations according to mNGS and blood-culture findings. The three-symbol classification indicates mNGS status / blood-culture status / antibiotic exposure at the time of blood/mNGS sampling (+ = positive/present; − = negative/absent). Abx+ and Abx− therefore refer exclusively to antimicrobial exposure status at sampling. Patients classified as Abx− could subsequently receive empirical antimicrobial therapy, which could later be escalated, de-escalated, or discontinued during the stewardship period. Treatment adaptations were categorized as escalation, de-escalation, or discontinuation.

mNGS / BC / Antibiotic	Underlying disease	Pathogens detected mNGS	Pathogens detected BC	Initial antimicrobial therapy before microbiological results	Antimicrobial stewardship change
**+ / - / +<br>Escalation**	Spondylodiscitis; sepsis	Epstein-Barr virus, Staphylococcus aureus, Human alpha herpesvirus 1 (HSV-1)	-	Flucloxacillin	+ Aciclovir
	Colonic anastomotic leak	Enterococcus faecium, Pseudomonas aeruginosa	-	Linezolid	+ Ciprofloxacin
	Duodenal perforation	Prevotella oris,Torque teno virus,Streptococcus spp.,Eikenella corrodens,Enterococcus faecium,Human cytomegalovirus,Pseudomonas mendocina	-	Meropenem	+ Penicillin G
	Gastric perforation	Epstein-Barr virus,Human alphaherpesvirus 1 (HSV-1)	-	Penicillin G	+ Aciclovir
	Pancreatic resection	Serratia spp.,Enterococcus faecalis	-	Ceftriaxone, Metronidazole	Ampicillin, Sulbactam
	Cecal perforation; peritonitis	Bacteroides ovatus,Klebsiella oxytoca,Alistipes finegoldii,Phocaeicola vulgatus,Odoribacter splanchnicus,Bacteroides thetaiotaomicron	-	Ceftriaxone	Piperacillin, Tazobactam
	Adhesive small-bowel obstruction;	Escherichia coli,Veillonella parvula,Streptococcus mutans,Streptococcus	-	Ceftriaxone, Metronidazole	Piperacillin, Tazobactam

	small-bowel perforation	anginosus,Haemophilus parainfluenzae			
	Sepsis; leg ulcer	Finegoldia magna,Prevotella bivia,Peptoniphilus harei,Schaalia odontolytica,Anaerococcus hydrogenalis,Anaerococcus lactolyticus,Streptococcus dysgalactiae	-	Ceftriaxone, Linezolid	+ Metronidazole
	Pyelonephritis	Escherichia coli	-	Ceftriaxone	Piperacillin, Tazobactam
	Anastomotic leak Colon	Enterobacter spp.,Enterococcus faecalis	-	Ceftriaxone, Metronidazole	Ciprofloxacin, Ampicillin, Sulbactam
	Pneumonia	Human cytomegalovirus	-		Ganciclovir
	Adhesion Ileus	Escherichia coli,Bacteroides caccae,Bacteroides ovatus,Morganella morganii,Phocaeicola vulgatus,Enterococcus faecalis	-	Meropenem	+ Ampicillin, Sulbactam
	NSTEMI	Human alphaherpesvirus 1 (HSV-1),Human betaherpesvirus 6B (HHV-6B)	-	Ampicillin, Sulbactam	+ Aciclovir
	Anastomotic leak	Escherichia coli,Enterococcus faecalis	-		Piperacillin, Tazobactam
	Joint infection	Enterobacter cloacae,Klebsiella pneumoniae,Pseudomonas aeruginosa	-		Piperacillin, Tazobactam
	Duodenal perforation	[Candida] glabrata,Enterococcus faecalis,Human cytomegalovirus,Human alphaherpesvirus 1 (HSV-1)	-	Anidulafungin	+ Ampicillin, Sulbactam
	Gastrointestinal bleeding	Escherichia coli,Human alphaherpesvirus 1 (HSV-1)	-	Meropenem	+ Aciclovir
	Peritonitis, abdominal abscess	Candida albicans,Enterococcus faecium,Streptococcus oralis,Aspergillus fumigatus	-	Meropenem, Vancomycin	+ Caspofungin
	Duodenal ischemia	Epstein-Barr virus,Enterococcus faecalis,Clostridium perfringens	-	Piperacillin, Tazobactam	Ampicillin, Sulbactam, Ciprofloxacin, Fluconazol
	Decompensated Heart failure	Helicobacter pylori	-	Ceftriaxone	+ Roxithromycin
	Peritonitis, perf. Cholecystitis	Klebsiella spp.,Proteus mirabilis,Citrobacter braakii,Morganella morganii,Clostridium perfringens,Citrobacter freundii complex sp. CFNIH3	-	Ceftriaxone, Metronidazole	+ Ciprofloxacin
	Pneumonia	Human cytomegalovirus	-	Cotrimoxazol	+ Aciclovir
	Pneumonia	Escherichia coli,Enterococcus faecium,Bacteroides uniformis,Sutterella wadsworthensis	-	Piperacillin, Tazobactam	Ampicillin, Sulbactam, Vancomycin
	Endocarditis	Human alphaherpesvirus 1 (HSV-1)	-	Piperacillin, Tazobactam	Aciclovir, Linezolid, Ceftriaxone

	Peritonitis	Enterococcus faecium,Pseudomonas aeruginosa	-	Ceftriaxone, Metronidazole	Piperacillin, Tazobactam, Vancomycin
	Gangrenous cholecystitis	Escherichia coli,Bacteroides fragilis		Ceftriaxone, Metronidazole	Piperacillin, Tazobactam
	Pneumonia, Influenza A pos.	Enterococcus faecium	-	Meropenem	
**+ / - / + De-escalation**	Duodenal leak	Enterococcus faecium,Human cytomegalovirus	-	Meropenem, Penicillin G, Anidulafungin	Ganciclovir
	Sepsis	Staphylococcus aureus	-	Flucloxacillin, Levofloxacin	Flucloxacillin
	Peritonitis	Hafnia spp.,Escherichia coli, Enterococcus durans, Enterococcus faecalis	-	Piperacillin, Tazobactam	Ampicillin, Sulbactam
	Urosepsis	Escherichia coli	-	Ceftriaxone, Metronidazole, Linezolid	Ceftriaxone, Linezolid
	Septic arthritis	Staphylococcus aureus	-	Clindamycin	Cefazolin
	Diabetic ketoacidosis	Streptococcus spp, Staphylococcus aureus	-	Piperacillin, Tazobactam	Ampicillin, Sulbactam
	Pneumonia	Mycoplasma pneumoniae	-	Ampicillin, Sulbactam, Roxithromycin	Levofloxacin
	Pneumonia	Haemophilus influenzae	-	Ampicillin, Sulbactam,	Ceftriaxone
	Pneumonia	Mycoplasma pneumoniae, Epstein-Barr virus	-	Ampicillin, Sulbactam, Roxithromycin	Doxacycline
	Pneumonia	Legionella pneumophila	-	Ciprofloxacin, Roxithromycin	Ciprofloxacin
	Pneumonia	Streptococcus pyogenes	-	Piperacillin, Tazobactam, Doxycycline, Cefazolin,	Penicillin G, Clindamycin
	Pneumonia	Streptococcus pneumoniae	-	Piperacillin, Tazobactam,	Ampicillin, Sulbactam
	Pleural Empyema	Streptococcus pneumoniae	-	Ampicillin, Sulbactam	Penicillin G
	Soft tissue infection	Epstein-Barr virus	-	Penicillin G, Clindamycin, Ganciclovir	Ganciclovir
	Endocarditis	Escherichia coli	-	Linezolid, Ceftazidim, Amphothericin	Linezolid, Ceftazidim
	Peritonitis	Escherichia coli	-	Meropenem, Vancomycin	Meropenem
	Pneumonia, Influenza A ST pos.	Enterococcus faecium		Meropenem	Enterococcus faecium was treated. Acyclovir for herpes labialis.
+ / - / + Stop	Post-operative soft tissue infection	[Candida] glabrata	-	Ceftriaxone, Metronidazole	
	Cardiac failure	Human betaherpesvirus 6B (HHV-6B)	-	Ampicillin, Sulbactam	
	Liver insufficiency	Epstein-Barr virus	-	Piperacillin, Tazobactam	

	Polytrauma	Enterococcus faecalis	-	Ceftriaxone, Metronidazole	
	Pneumonia	Epstein-Barr virus	-	Piperacillin, Tazobactam	
	Mechanical ileus	Enterococcus faecalis,Human betaherpesvirus 6B (HHV-6B)	-	Ceftriaxone, Metronidazole	
	Pneumonia	Epstein-Barr virus	-	Ceftrixone	
	Hernia incarcerated	Epstein-Barr virus	-	Anidulafungin	
	Ileus	Human betaherpesvirus 6B (HHV-6B)	-	Ceftriaxone, Metronidazole	
+ / + / + Escalation	Anastomotic leak	Hafnia paralvei, Enterococcus faecium, Bacteroides eggerthii, Staphylococcus aureus, Flavonifractor plautii, Akkermansia muciniphila, Parabacteroides goldsteinii, Bacteroides thetaiotaomicron	S. epidermidis	Ceftriaxone, Metronidazole	Vancomycin
**+ / + / + De-escalation**	Pneumonia	Legionella pneumophila	S. epidermidis	Ampicillin, Sulbactam	Levofloxacin
	Sepsis	Epstein-Barr virus, Streptococcus pyogenes	S. epidermidis	Meropenem	Penicillin G
	Pancreatitis	Staphylococcus aureus, Human alphaherpesvirus 1 (HSV-1)	S. hominis	Meropenem	Co-Trimoxacol
	pAVK IV	Staphylococcus aureus	Staphylococcus aureus	Piperacillin, Tazobactam Clindamycin	Flucloxacillin
	Sepsis	Streptococcus pyogenes	Staphylococcus epidermidis	Meropenem	Penicillin G
**+ / - / - Escalation**	Stomach perforation	Clostridium perfringens	-	-	Penicillin G
	Sigma perforation	Human alphaherpesvirus 1 (HSV-1)	-	-	Aciclovir
	Pyelonephritis	Escherichia coli	-	-	Cefuroxim
	Pyelonephritis	Escherichia coli, Lactobacillus amylovorus	-	-	Ceftriaxone, Metronidazole, Linezolid
	Anastomotic leak	Epstein-Barr virus, Varizella-Zoster-Virus (HSV-3)	-	-	Aciclovir
	Pyelonephritis	Escherichia coli	-	-	Meropenem
	Pyelonephritis	Escherichia coli	-	-	Piperacillin, Tazobactam
	Peritonitis	Enterococcus faecalis	-	-	Vancomycin
	Mechanical ileus	Escherichia coli,Human alphaherpesvirus 1 (HSV-1)	-	-	Ceftriaxone, Metronidazole
	Decompensated heart failure	Enterobacter cloacae, Human cytomegalovirus	-	-	Ciprofloxacin
	Cholangitis	Klebsiella spp., Escherichia coli, Epstein-Barr virus, Clostridium perfringens, Human alphaherpesvirus 1 (HSV-1)	-	-	Piperacillin, Tazobactam
	Peritonitis	Escherichia coli, Clostridium paraputrificum	-	-	Piperacillin, Tazobactam
	Mechanical ileus	Klebsiella spp., Enterococcus spp., Epstein-Barr virus	-	-	Ceftriaxone

	Cecal carcinoma, mechanical ileus	Phocaeicola spp., Alistipes finegoldii, Bacteroides uniformis, Bacteroides cellulosilyticus	-	-	Ceftriaxone, Metronidazole
	Phlegmon	Varizella-Zoster-Virus (HHV-3)	-	-	Aciclovir
	Endocarditis	Enterococcus faecalis, Torque teno virus 22 (TTV-22)	-	-	Ampicillin, Ceftriaxone
	Pneumonia	Klebsiella spp.	-	-	Meropenem
	Sepsis/ Femoral neck fracture	Enterococcus faecium	-	-	Meropenem
	Gastrointestinal perforation	Staphylococcus epidermidis	-	-	Ceftriaxone, Metronidazole
	Decompensated heart failure, renal failure, liver failure.	Enterobacter spp.,Human cytomegalovirus	-	-	Ciprofloxacin
	Respiratory infection / pneumonia	Streptococcus spp.,Human betaherpesvirus 6A (HHV-6A)	-	-	Piperacillin, Tazobactam
			-	-	
+ / - / - De-escalation	Anastomotic leak	Enterococcus faecalis, Prevotella melaninogenica	-	Ceftriaxone, Metronidazole	Ampicillin, Sulbactam
	Pneumonia	Prevotella oris, Staphylococcus aureus, Streptococcus constellatus	-	Piperacillin, Tazobactam	Ampicillin, Sulbactam
	Pneumonia	Haemophilus influenzae, Streptococcus pneumoniae	-	Piperacillin, Tazobactam, Clarithromycin	Penicillin G, Ceftriaxone
	Peritonitis	Haemophilus parainfluenzae	-	Ceftriaxone, Metronidazole	Caspofungin (Candida albicans detected in an abdominal swab)
	Sepsis, splenic rupture	Staphylococcus aureus	-	Ceftriaxone, Metronidazole	Ceftriaxone
	Pneumonia	Streptococcus pneumoniae		Piperacillin, Tazobactam	Ampicillin, Sulbactam
		Staphylococcus epidermidis		Vancomycin / Ampicillin, Sulbactam	Cefazolin
+ / + / - Escalation	Soft tissue infection	Escherichia coli, Enterococcus faecium	E. coli E. faecium		Ceftriaxone, Vancomycin
	Duodenal leak	Enterobacter spp., Enterococcus spp., Paeniclostridium sordellii	Enterobacter absuriae		Ciprofloxacin, Metronidazole
	Stomach perforation	Enterococcus faecium	Corynebacterium striatum		Vancomycin
	Pneumonia	Escherichia coli, Phocaeicola vulgatus, Bacteroides uniformis, Pseudomonas aeruginosa, Sutterella wadsworthensis, Bacteroides thetaiotaomicron	S. aureus		Piperacillin, Tazobactam Tobramycin
	Pneumonia, Aspiration	Lactococcus lactis, Streptococcus thermophilus	Cutibacterium spp.		Piperacillin, Tazobactam
	Pneumonia	Escherichia coli	E. coli		Ampicillin, Sulbactam

	Renal failure	Escherichia coli, Klebsiella pneumoniae	E. coli, Klebsiella pneumoniae		Piperacillin, Tazobactam
	Bronchial carcinoma	Enterococcus faecium	S. epi, S. hominis		Linezolid
					8
**+ / + / - De-escalation**	Cholangiosepsis	Escherichia coli, Enterobacter cloacae, Enterobacter hormaechei, Haemophilus parainfluenzae	Enterbacter cloacae	Meropenem	Ciprofloxacin
	Peritonitis	Escherichia coli	E. coli	Meropenem, Vancomycin	Ciprofloxacin
	Pneumonia	Epstein-Barr virus, Streptococcus pneumoniae	S. pneumoniae	Ampicillin, Sulbactam, Roxithromycin	Penicillin G
	Endocarditis	Streptococcus gordonii	S. gordonii	Ampicillin, Sulbactam	Penicillin G
	Pneumonia	Streptococcus pneumoniae	S. pneumoniae	Ampicillin, Sulbactam, Clarithromycin Penicillin G	Penicillin G
	Sepsis, Intra-abdominal infection / perforation	Proteus vulgaris,Epstein-Barr virus,Enterococcus faecium,Enterococcus faecalis,Klebsiella pneumoniae	Staphylococcus epidermidis,	Meropenem Vancomycin Anidulafungin	Meropenem Vancomycin
**- / - / + De-escalation**	Stomach perforation	-	-	Clarithromycin, Metronidazole, Vancomycin, Voriconazol, Aciclovir	- Vancomycin
	Soft tissue infection	-	-	Piperacillin, Tazobactam, Clindamycin	- Clindamycin
**- / - / + Escalation**	Pneumonia	-	-	Ampicillin, Sulbactam, Roxithromycin	Piperacillin, Tazobactam
**- / - / + Stop**	Pneumonia	-	-	Levofloxacin, Ceftriaxone, Fluconazol	
	Staphylococcal scale skin syndrome	-	-	Ceftriaxone, Fluconazol	
	Peritonitis	-	-	Piperacillin, Tazobactam, Amphothericin B	
	Sepsis, Ulcus cruris	-	-	Ampicillin, Sulbactam, Levofloxacin	
	Pyelonephritis	-	-	Meropenem	
	Stomach perforation, Peritonitis	-	-	Ceftriaxone, Metronidazole	
	Pneumonia, Covid-19 positive	-	-	Piperacillin, Tazobactam	
	Soft tissue infection	-	-	Ampicillin, Sulbactam, Levofloxacin	

	Infection of unknown origin	-	-	Ampicillin, Sulbactam	
	Duodenal resection	-	-	Ceftriaxone, Metronidazole	
	Cardiac decompensation	-	-	Amoxicillin, Clavulanic acid	
	Hip fracture	-	-	Piperacillin, Tazobactam	
**- / + / + De-escalation**	Sepsis, Ulcus cruris	-	S. haemolyticus	Piperacillin, Tazobactam, Metronidazole	Levofloxacin

Selected detailed examples of episode-level antimicrobial treatment adaptations according to mNGS and blood-culture findings.
